# Crystal structures of sodium-, lithium-, and ammonium 4,5-di­hydroxy­benzene-1,3-di­sulfonate (tiron) hydrates

**DOI:** 10.1107/S2056989018008009

**Published:** 2018-06-08

**Authors:** Corey J. Herbst-Gervasoni, Michael R. Gau, Michael J. Zdilla, Ann M. Valentine

**Affiliations:** a Department of Chemistry, Temple University, 1901 N. 13th St., Philadelphia, PA 19122, USA; b Department of Chemistry, University of Pennsylvania, 231 S. 34 Street, Philadelphia, PA 19104, USA

**Keywords:** bioinorganic, catechol, metal organic framework, sodium channel, crystal structure

## Abstract

The solid-state structures of the Na^+^, Li^+^, and NH_4_^+^ salts of the 4,5-di­hydroxy­benzene-1,3-di­sulfonate (tiron) dianion are reported, namely disodium 4,5-di­hydroxy­benzene-1,3-di­sulfonate, 2Na^+^·C_6_H_4_O_8_S_2_^2−^, μ-4,5-di­hydroxy­benzene-1,3-di­sulfonato-bis­[aqua­lithium(I)] hemihydrate, [Li_2_(C_6_H_4_O_8_S_2_)(H_2_O)_2_]·0.5H_2_O, and di­ammonium 4,5-di­hydroxy­benzene-1,3-di­sulfonate monohydrate, 2NH_4_^+^·C_6_H_4_O_8_S_2_^2−^·H_2_O. Inter­molecular inter­actions vary with the size of the cation, and the asymmetric unit cell, and the macromolecular features are also affected. The sodium in Na_2_(tiron) is coordinated in a distorted octa­hedral environment through the sulfonate oxygen and hydroxyl oxygen donors on tiron, as well as an inter­stitial water mol­ecule. Lithium, with its smaller ionic radius, is coordinated in a distorted tetra­hedral environment by sulfonic and phenolic O atoms, as well as water in Li_2_(tiron). The surrounding tiron anions coordinating to sodium or lithium in Na_2_(tiron) and Li_2_(tiron), respectively, result in a three-dimensional network held together by the coordinate bonds to the alkali metal cations. The formation of such a three-dimensional network for tiron salts is relatively rare and has not been observed with monovalent cations. Finally, (NH_4_)_2_(tiron) exhibits extensive hydrogen-bonding arrays between NH_4_^+^ and the surrounding tiron anions and inter­stitial water mol­ecules. This series of structures may be valuable for understanding charge transfer in a putative solid-state fuel cell utilizing tiron.

## Abstract 2

The first solid-state structures of the Na^+^, Li^+^, and NH_4_^+^ salts of the tiron dianion are reported. The structures reveal significant changes in local and long-range ionic inter­actions with variation of the cation, with consequences for fields as disparate as bioinorganic chemistry and fuel cell technologies.

## Chemical context   

Catechols play important roles across many areas of chemistry and biology. Their rich coordination chemistry with metal ions (Pierpont & Lange, 1994 ▸; Sever & Wilker, 2004 ▸) emerges for example in siderophores (Boukhalfa & Crumbliss, 2002 ▸; Raymond *et al.*, 2015 ▸; Springer & Butler, 2016 ▸). One catechol-containing siderophore, enterobactin (ent) has the strongest characterized Fe^III^ complex to date (*K*_a_ = 10^49^) (Loomis & Raymond, 1991 ▸). Catechols are also key to the function of some marine bioadhesives (Lee *et al.*, 2011 ▸); in one recent example, a protein in sessile marine organisms uses a cooperation between surface residues containing 3,4-di­hydroxy­phenyl­alanine (DOPA) and lysine to bind strongly to mineral surfaces (Rapp *et al.*, 2016 ▸). Some species of ascidians produce a polyphenol-containing mol­ecule called tunichrome that has been implicated in metal binding and/or metal function (Sugumaran & Robinson, 2012 ▸).

**Figure 1:**
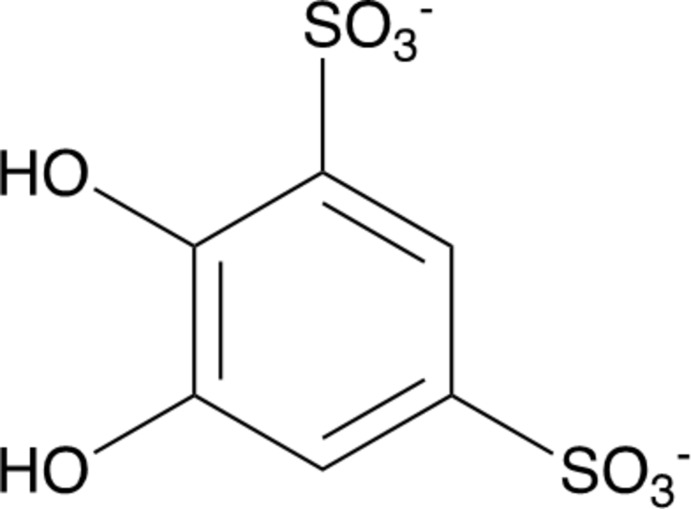
Tiron dianion (4,5-dihy­droxy-1,3-benzene­disulfonate).

Upon binding to metal cations such as Fe^III^ and Ti^IV^, cat­echols typically form brightly colored complexes (Sever & Wilker, 2004 ▸; Pierpont & Lange, 1994 ▸). In solution, however, some catechols can oxidize and form polymers, thus forming metal complexes that are more difficult to characterize. Compared to unsubstituted catechol, tiron (4,5-dihy­droxy-1,3-benzene­disulfonic acid, Fig. 1 ▸) allows for improved water solubility as well as reduced polymerization by substituting electron-withdrawing sulfonic acid moieties (Sommer, 1963*a* ▸,*b* ▸). Tiron has long been used for colorimetric determination of both Ti^IV^ and Fe^III^ (Yoe & Armstrong, 1945 ▸, 1947 ▸), hence its name.

The free acid of tiron has been used in an aqueous flow battery because of its two-electron redox couple within range of an aqueous system, high water solubility, and low cost (Yang *et al.*, 2014 ▸). When crystallized, tiron mol­ecules can form a network through coordination of the counter-cation to the sulfonate or protonated or deprotonated hydroxide of the tiron (Côté & Shimizu, 2001 ▸, 2003 ▸; Sheriff *et al.*, 2003 ▸; Guan & Wang, 2016 ▸, 2017 ▸). These networks can range from one-dimensional networks, which form a linear polymer (Côté & Shimizu, 2003 ▸; Sheriff *et al.*, 2003 ▸), to three-dimensional networks in which each tiron anion is coordinated to a metal cation and forms an inter­connected lattice among all tiron anions in the crystal (Côté & Shimizu, 2001 ▸, 2003 ▸; Guan & Wang, 2016 ▸). Many of these tiron-containing crystal structures exhibit counter-cation-dependent luminescent properties (Guan & Wang, 2016 ▸, 2017 ▸). The three-dimensional networks with tiron can absorb H_2_S gas after inter­stitial and coordinated H_2_O are liberated with heat (Côté & Shimizu, 2003 ▸). Currently, examples of three-dimensional networks formed by tiron and cations are relatively rare. Presented here are the first two examples of the preparation and characterization of the Li^+^ tiron salt and Na^+^ tiron salt, which forms a three-dimensional network. In addition to Li_2_(tiron) and Na_2_(tiron), the preparation and crystallization of the NH_4_^+^ tiron salt is reported. This species is the first tiron salt which utilizes a counter-cation capable of hydrogen bond (H-bond) donation to allow for a complex H-bonding network.
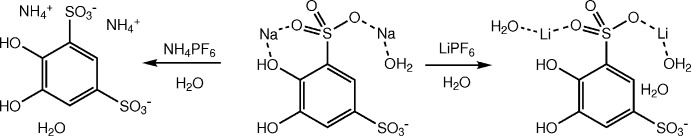


## Structural commentary   

Three tiron salts of different monovalent cations were crystallized. Li_2_(tiron) and (NH_4_)_2_(tiron) were both prepared from commercially available Na_2_(tiron) by salt metathesis. In each case, the Na^+^ cation was removed by 15-crown-5 ether.

**Figure 2:**
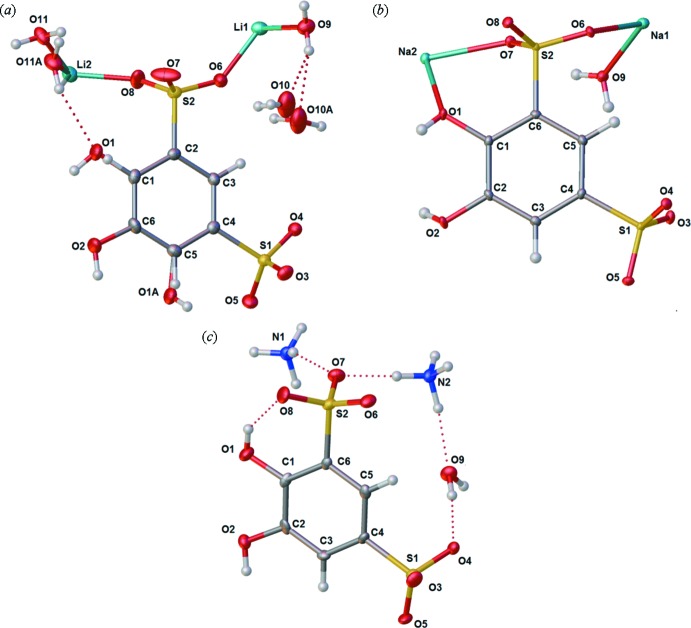
Displacement ellipsoid plots of the asymmetric unit contents for the crystal structures of tiron salts characterized in this study: (*a*) Li_2_(tiron)·2.5H_2_O, (*b*) Na_2_(tiron)·H_2_O and (*c*) (NH_4_)_2_(tiron)·H_2_O. Ellipsoids are shown at the 50% probability. Hydrogen atoms shown as spheres.

All asymmetric units (Fig. 2 ▸) contain two of their respective cations on general positions. Water is included in all asymmetric units, however in different amounts. Both sodium and ammonium tiron have one water mol­ecule in the asymmetric unit, whereas lithium tiron has 2.5 water mol­ecules in the asymmetric unit. The lithium tiron also exhibits rotational whole-mol­ecule disorder leading to two possible placements of O1 on the phenyl, and representing a major and minor orientation [89.2 (3) and 10.8 (3)% occupancy, respectively].

**Figure 3:**
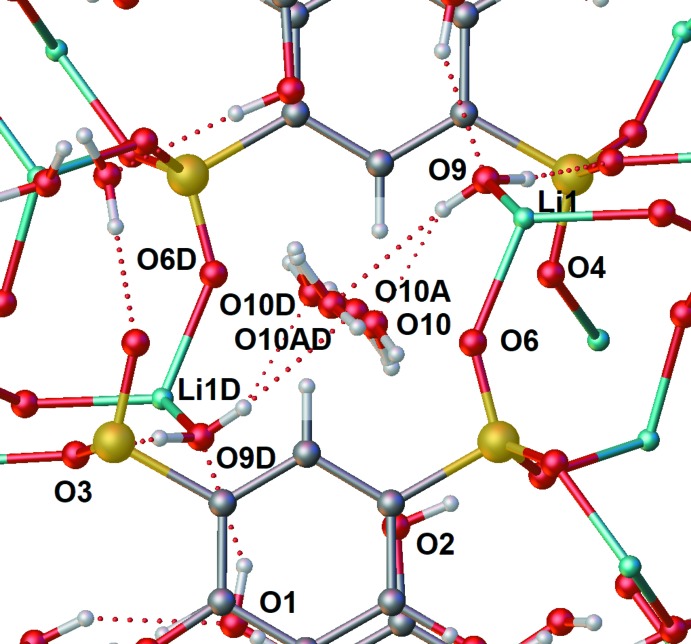
Ball and stick representation of Li_2_(tiron)·2.5H_2_O, including the H-bonding environment of neighboring tiron anions and water showing all disorder components. Atom labels with the suffix D are generated *via* inversion through the center of symmetry at Wyckoff position *d*. Atom labels with the suffix A represent minor components of two-site disorder models.

The structure of Li_2_(tiron) is presented in the *P*2_1_/*n* space group. The lithium ion is coordinated by phenolic, sulfonate and water oxygen atoms. Lithium is bonded to only three sulfonate moieties, and one water mol­ecule in a distorted tetra­hedral geometry. An extensive H-bonding network with three types of solvate water mol­ecules stabilizes the crystal structure (Table 1 ▸, Fig. 3 ▸). The geometrically frustrated water mol­ecule containing O10 sits in a pocket surrounded by H-bond donors and acceptors from sulfonate (O6, O4), and water (O9), and phenol (O2). As a result of the frustration, O10 is highly disordered, modeled with a two-site split-atom model that additionally exhibits special-position disorder about the inversion element at Wyckoff position *d*. The result is a four-site disorder model for the water mol­ecule containing O10. The lithium-bound water mol­ecule containing O11 H-bonds with sulfonate oxygen atoms O4 and O8, but the oxygen atom is disordered, pyramidalized predominantly toward the phenolic O—H hydrogen atom of O2 due to H-bonding, but with a minor component pyramidalized toward O1, which is less available as an H-bond acceptor since the phenolic hydrogen of O1 is already involved in an intra­molecular *ortho*-H-bond with its own O2 (Fig. 2 ▸). The water mol­ecule containing O9 is also lithium bound, but not disordered, and inter­acts with O10/10*A* of the disordered water and with sulfonate oxygen O3 and the phenolic hydrogen atom of O1.

**Figure 4:**
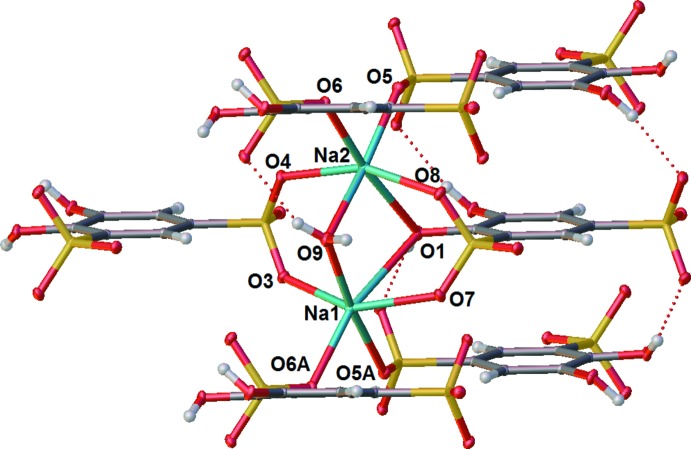
Displacement ellipsoid plot of Na_2_(tiron)·H_2_O illustrating the pseudo-octa­hedral coordination geometry around the Na ions. Ellipsoids shown at the 50% probability level. H atoms are shown as spheres. Atom labels with the suffix A are related by translation by one unit cell.

The sodium salt of tiron is also presented in the *P*2_1_/*n* space group. Each sodium atom is bonded to four sulfonate moieties, one hydroxide, and one water oxygen atom to give a distorted octa­hedral geometry (Fig. 4 ▸). The two types of Na atoms are bridged to one another along the crystallographic *a* axis by O9 of a water ligand and by phenolic oxygen residue O1 on one side, and by sulfonate residues O6 and O5 on the other.

**Figure 5:**
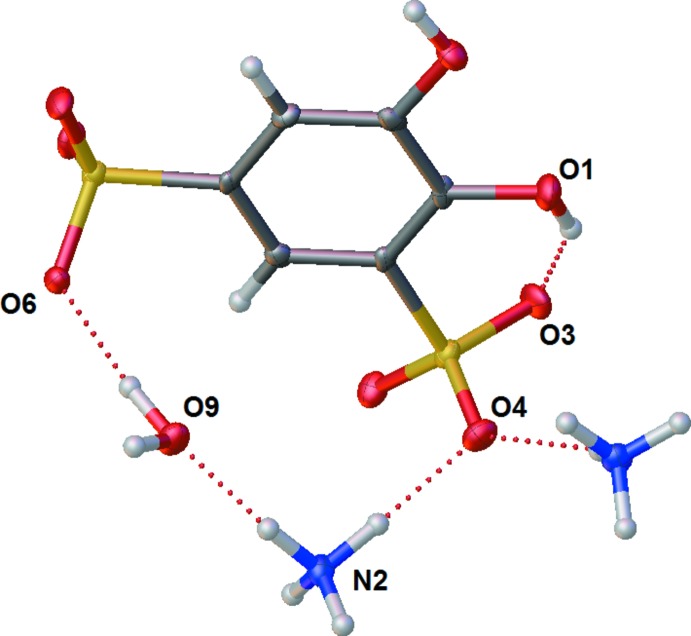
Displacement ellipsoid plot of asymmetric-unit contents for the crystal structure of (NH_4_)_2_(tiron)·H_2_O, showing intra­molecular H-bonding with ammonium ions and solvate water. Ellipsoids shown at the 50% probability level. Hydrogen atoms shown as spheres.

Finally, (NH_4_)_2_(tiron) is presented in the *Pbca* space group. Ammonium is oriented around the negatively charged sulfonates, and acts as an H-bond donor to both sulfonates and neighboring water mol­ecules (Table 3 ▸, Fig. 5 ▸). The structure of (NH_4_)_2_(tiron) is well-ordered with a clear H-bonding network, discussed in more detail in the next section.

## Supra­molecular features   

**Figure 6:**
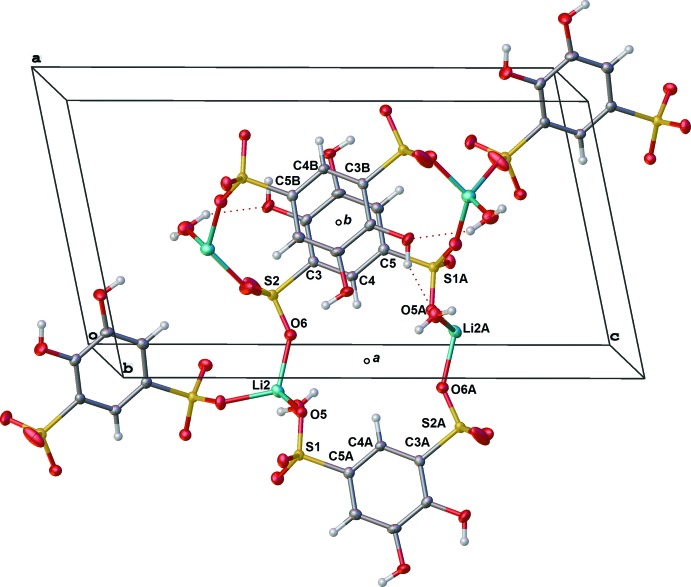
Illustration of selected nearest neighbor lithium linkages of neighboring tiron anions in Li_2_(tiron)·2.5H_2_O. Atom labels with the suffix B are generated *via* inversion through the center of symmetry at the center of the cell (Wyckoff *b*) and those with the suffix A are generated *via* inversion through the center of symmetry in the *a*-face (Wyckoff *b*). Ellipsoids shown at the 50% probability level. Hydrogen atoms are shown as spheres.

All three tiron salts exhibit π-stacking between tiron catechol moieties, augmented by H-bonding inter­actions. H-bonding is present inter- and intra­molecularly for all tiron salts in this study. The lithium salt exists in the solid state as a three-dimensional inter­connected array of tiron anions bridged by lithium ions (Fig. 6 ▸). One of the lithium ions, Li1 serves to bridge two sulfonate groups of two neighboring tiron arenes, which π-stack with one another about a crystallographic inversion center (Wyckoff position *b*), hence the rings are perfectly parallel. The other lithium ion, Li2, links through a sulfonate group (S1) of one tiron to the other sulfonate group S(2) of a third tiron, generating a ‘square’ assembly of tiron anions and two Li2 ions around a crystallographic inversion element (Wyckoff position *a*, Fig. 6 ▸). The distance of 3.718 (10) Å between the centroids of neighboring arene rings is consistent with a strong π-stacking inter­action, and suggests the inter­action is augmented by the array of H-bonding inter­actions among phenolic hydroxyl and sulfonate groups and water (Table 1 ▸, Fig. 3 ▸).

**Figure 7:**
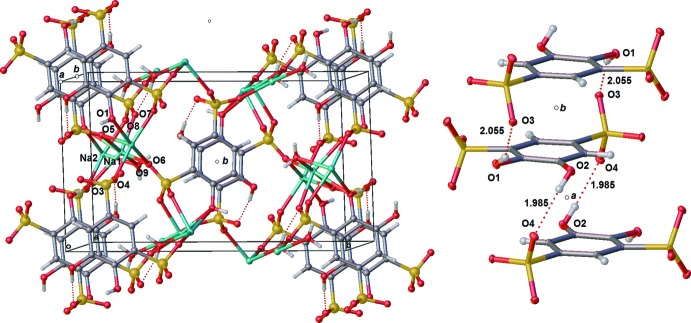
Packed structures of Na_2_(tiron)·H_2_O. Left: packing arrangement in the unit cell. Right: displacement ellipsoid plot showing alternating units in a π-stacked arrangement in the crystallographic *a-*axis direction. Ellipsoids are shown at the 50% probability level, hydrogen atoms are shown as spheres.

The H-bonding in the sodium complex is entirely inter­molecular (Table 2 ▸, Fig. 7 ▸). Both hydroxyl moieties H-bond to a sulfonate moiety on an adjacent tiron anion. The hydroxyl O1 H-bonds to the sulfonate based O3 [O—H⋯O—S 2.05 (2) Å]. The other hydroxyl O4 H-bonds to O2 of the same sulfonate with a slightly shorter H-bond [O—H⋯O—S 1.98 Å]. These two H-bonds decrease hyperconjugation to the π-system from the oxygen atom in the hydroxyls by reducing the torsion angle by 32.08 and 46.16° for O1 and O2, respectively. This deviation from a fully hyperconjugated hydroxyl exemplifies the importance of the formation of the H-bond. An H-bond not shown exists between a proton in water bound by Na^+^ and a tiron-based hydroxyl O2 position as well as a sulfonate in the first position [O—H⋯O—H 2.18 (3) Å, O—H⋯O—S 2.14 (3) Å].

**Figure 8:**
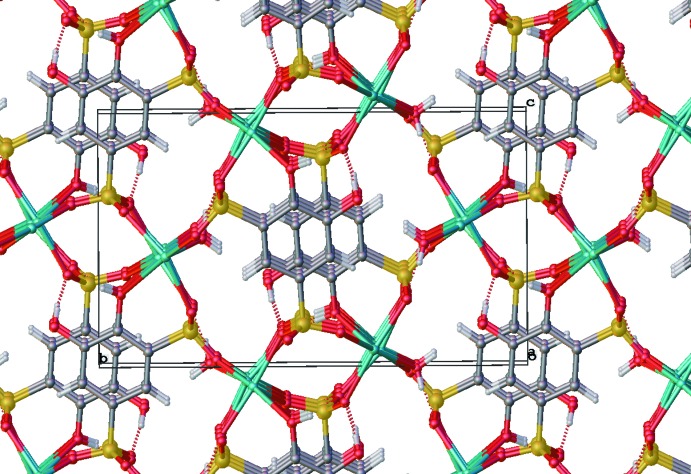
Extended packing diagram of Na_2_(tiron)·H_2_O, showing the columnar arrangement of tiron aryl groups and parallel sodium ion channels.

Two types of sodium atoms arrange in channels along the crystallographic *a*-axis direction, and are bridged by sulfonyl oxygen atoms O3, O4, O5, and O6, by phenolic oxygen atom O1, and by water oxygen atom O9 (Fig. 7 ▸). Neighboring tiron arenes π-stack along the crystallographic *a*-axis direction, related by crystallographic inversion centers (Wyckoff letters *a* and *b*), also requiring the arene rings to be parallel, as in the lithium salt. The π-stacking inter­actions are further augmented by H-bonding inter­actions between the phenolic hydrogens of O1 and O2 with sulfonate oxygens O3 and O4 respectively. The π-stacking distance of 3.753 (18) Å is similar to that observed in the lithium salt with its corresponding dense array of H-bonding inter­actions, but slightly shorter due to the more acute O—Na—O bond angles in octa­hedrally coordinated sodium atoms, in contrast to tetra­hedrally coord­inated lithium atoms in the lithium salt. This arrangement of Na^+^ and tiron ions results in an ordered array of sodium channels inter­spersed between columns of strongly inter­acting π-stacked tiron aryl groups, all in the crystallographic *a*-axis direction (Fig. 8 ▸).

**Figure 9:**
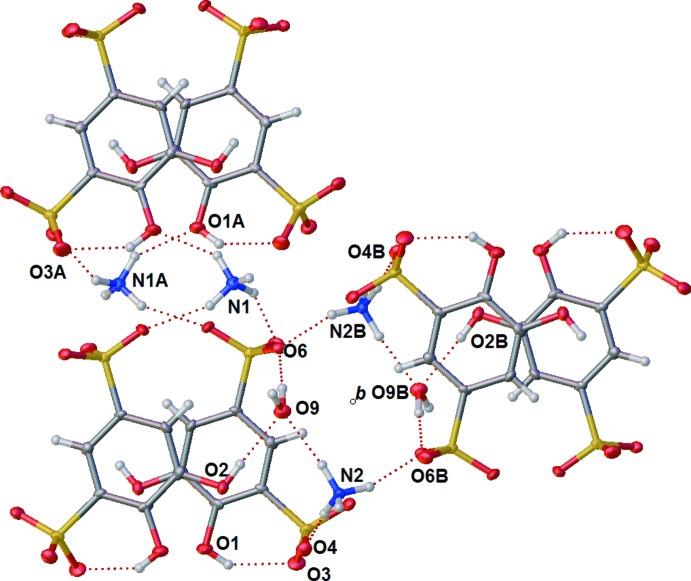
Displacement ellipsoid plot of (NH_4_)_2_(tiron)·H_2_O showing neighboring H-bonding inter­actions among six tiron anions, four ammonium ions, and two water solvate mol­ecules. Ellipsoids are shown at the 50% probability level and hydrogen atoms are shown as open spheres. Atom labels with the suffix B are generated by symmetry about a crystallographic center of inversion (Wyckoff position *b*), and atom labels with the suffix A are generated by one or more glide operation.

Unlike Na^+^ and Li^+^, NH_4_^+^ cannot be coordinated by any atoms on tiron or water. Because of this inability, NH_4_^+^ inter­actions with the surrounding mol­ecules are primarily H-bond based. Both ammonium ions H-bond to three sulfonate moieties and an oxygen atom from a phenolic hydroxyl or a water molecule (Tables 3 ▸ and 4 ▸, Fig. 9 ▸). The ammonium ion containing N1 forms H-bonds with two tiron mol­ecules that are horizontally next to each other in the unit cell as well as a tiron above and a tiron below. The ammonium ion containing N2 also forms a similar H-bonding network with the tiron mol­ecules but also stabilizes an inter­stitial water. This water H-bonds to two first position sulfonate moieties in alternating layers of tiron mol­ecules [O—H⋯O—S 1.97 (2) Å, O—H⋯O—S 2.03 (2) Å]. Finally, O5 of a phenolic hydroxyl is H-bonded to this inter­stitial water [O—H⋯OH_2_ 1.99 Å] (Fig. 9 ▸). Regarding intra­molecular H-bonding, because the protons on the hydroxyls are pointed away from each other to allow for H-bonding to N1, the phenolic hydroxyl containing O1 is directed to H-bond with O3 of the sulfonate (Fig. 9 ▸).

**Figure 10:**
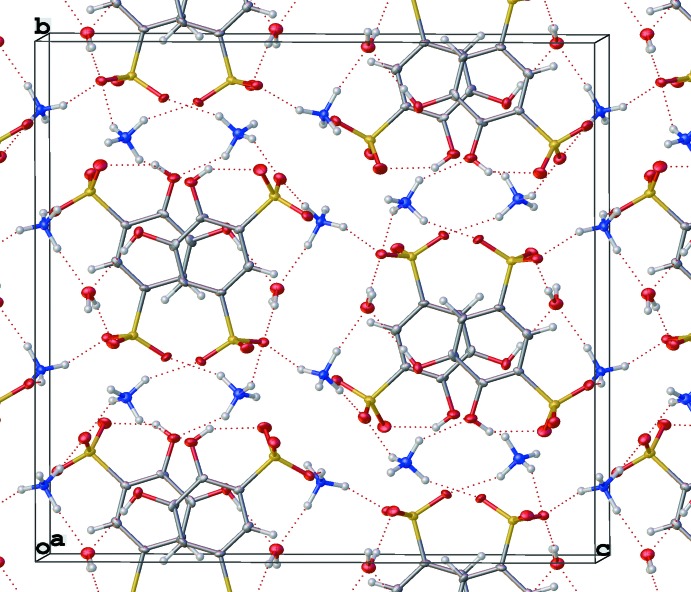
Extended packing view of (NH_4_)_2_(tiron) structure showing the three-dimensional H-bond network.

The (NH_4_)_2_(tiron) packs such that the tiron units connect to one another along the crystallographic *c*-axis direction *via* a six-membered H-bonding array of two lattice water mol­ecules, two ammonium ions (containing N2), and two sulfonate oxygen atoms (Fig. 9 ▸). The ammonium ions containing N1 further serve to link tiron units along the crystallographic *b*-axis direction by H-bonding with sulfonate oxygen O8 and phenolic oxygen atom O1. Further, the arene π-stacking inter­actions and additional H-bonding inter­actions between sulfonate oxygen atoms and both ammonium ions link these strands to one another in the crystallographic *a*-axis direction to give the three-dimensional H-bonding network (Fig. 10 ▸), although the π-stacking distance is greatest in the ammonium salt [4.006 (3) Å] in comparison to the Li^+^ and Na^+^ salts. This result is possibly due to the lower strength of N—H H-bonds in comparison to O—H H-bonds. Unlike the Li^+^ and Na^+^ salts, the planes of the arene rings of tiron in the NH_4_^+^ tiron are canted at an angle of 2.08°, related to one another by the crystallographic glide operations.

## Database survey   

In the reported structures, inter­actions with sulfonate moieties and the protonated hydroxyl moieties together create a complex network formed through coordinate bonds or H-bonds. A search of the Cambridge Structural Database (Version 5.39, February 2018; Groom *et al.*, 2016 ▸) yielded several structures that included tiron (Table 5 ▸). Of the structures reported, seven exhibited π-stacking inter­actions between at least two tiron mol­ecules as represented by their inter­centroid distances. A rarer structural feature of these complexes is the formation of networks between tiron mol­ecules and their corresponding counter-cations in which only HUCMOH, ADOXUP, and HUCMOH02 form three-dimensional networks by eliciting multiple bonds to the cations (Côté & Shimizu, 2003 ▸; Guan & Wang, 2016 ▸). Both presented Li^+^ and Na^+^ tiron salts are the first examples of tiron-containing structures with monovalent cations that form three-dimensional networks. Furthermore, the NH_4_^+^ tiron salt presented is the first example of a tiron complex in which the counter-cation H-bonds to the tiron.

## Synthesis and crystallization   

*Na_2_(tiron)·H_2_O*

Na_2_(tiron)·H_2_O was used as received from the commercial source (97%, Sigma Aldrich) and added to water until it was saturated. The slurry was filtered into a 1 dram scintillation vial and covered with a Kimwipe. The solution was allowed to sit undisturbed at room temperature until the water evapor­ated. The crystals which developed were off-white needles.

*Li_2_(tiron)·2.5H_2_O*

In 2.00 mL of water, 0.100 g of Na_2_(tiron)·H_2_O was dissolved. To this solution, 0.94 g of LiPF_6_ was added. Once the lithium salt dissolved, 0.120 mL 15-crown-5 was added which immediately resulted in a white precipitate. The slurry was stirred while adding 1 mL of dichloromethane (DCM), then the DCM layer was removed by pipette. The aqueous solution was extracted twice more with DCM for a total of three times. The water was then evaporated by gentle heating. A white powder was obtained and triturated with 1 mL of diethyl ether three times. The resulting solid was dried, partially dissolved in ethanol, filtered, and allowed to sit undisturbed in a 1 dram scintillation vial at room temperature. The resulting crystals were off-white needles.

*(NH_4_)_2_(tiron)·H_2_O*

Na_2_(tiron)·H_2_O (0.100 g) was added and dissolved in 2.00 mL of water. After the Na_2_(tiron) had dissolved, 0.098 g of NH_4_PF_6_ was added and dissolved. Upon adding NH_4_PF_6_, the solution turned rose pink. To this solution, 0.120 mL of 15-crown-5 was added and a white precipitate formed. The slurry was extracted three times total with 1 mL of DCM each time. The aqueous solution was gently heated to dryness. The solid was then triturated with three separate 1 mL portions of diethyl ether. The solid was dried and dissolved in methanol, filtered, and allowed to sit undisturbed in a 1 dram vial at room temperature. The crystals which formed were off-white needles with a purple/rose-colored oily residue coating them.

## Refinement   

Crystal data, data collection and structure refinement details are summarized in Table 6 ▸. Water hydrogens were located in difference maps and refined wherever possible. H atoms bonded to C were placed in geometrically idealized positions based on *sp* ^2^ hybridization with C—H bond lengths of 0.95 Å and *U*_iso_(H) = 1.2*U*_eq_(C). A combination of calculated H atoms and H atoms found in the difference map was utilized for phenolic O—H and H_2_O mol­ecules. For phenolic OH, H atoms were either located and freely refined, or placed in idealized *sp* ^3^ positions with bond lengths of 0.84 Å and *U*_iso_(H) = 1.5*U*_eq_(O), and permitted to rotate about the O—C bond. For water mol­ecules, hydrogen atoms were either located in the difference-Fourier map and refined with restraints (detailed below), or added and refined with restraints according to the most likely hydrogen-bonding inter­actions. For disordered water mol­ecules, restraints (SIMU, DFIX, DANG, ISOR) and constraints (EADP) were employed to improve displacement parameters as well as allow for convergence of H-atom locations. For SIMU restraints on the disordered O10 of Li_2_(tiron)·2.5H_2_O, the restraint was set at s = 0.005 Å, st = 0.02 Å, and the default cutoff of 1.7 Å. These atoms were further restrained with ISOR 0.01 0.02. The disordered O—H phenoxyl group on the tiron ligand for the Li_2_(tiron) crystal structure was located from a difference map and refined to a 0.892 (3)/0.108 (3) site occupancy. The lithium-bound water mol­ecule was refined to a 0.751 (12)/0.249 (12) site occupancy. The disordered solvate water mol­ecule on Wyckoff position *d* containing O10 was refined to a 0.30 (5)/0.20 (5) site occupancy. H atoms bonded to N were located in the difference-Fourier maps and restrained using DFIX and DANG to idealized *sp* ^3^ hybridization with N—H bond lengths of 1.00 (2) Å and *U*_iso_(H) = 1.5*U*_eq_(N). EADP was used to constrain the ellipsoids of the disordered O1 to be equivalent for accurate refinement of occupancies.

## Supplementary Material

Crystal structure: contains datablock(s) global, NaTiron, NH4Tiron, LiTiron. DOI: 10.1107/S2056989018008009/zl2730sup1.cif

Structure factors: contains datablock(s) NaTiron. DOI: 10.1107/S2056989018008009/zl2730NaTironsup2.hkl

Structure factors: contains datablock(s) NH4Tiron. DOI: 10.1107/S2056989018008009/zl2730NH4Tironsup3.hkl

Structure factors: contains datablock(s) LiTiron. DOI: 10.1107/S2056989018008009/zl2730LiTironsup4.hkl

Supporting information file. DOI: 10.1107/S2056989018008009/zl2730NaTironsup5.cdx

Supporting information file. DOI: 10.1107/S2056989018008009/zl2730NH4Tironsup6.cdx

Supporting information file. DOI: 10.1107/S2056989018008009/zl2730LiTironsup7.cdx

CCDC references: 1811385, 1811387, 1811386

Additional supporting information:  crystallographic information; 3D view; checkCIF report

## Figures and Tables

**Table 1:** Hydrogen-bond geometry (Å, °) for Li_2_(tiron)·2.5H_2_O[Chem scheme1]

*D*—H⋯*A*	*D*—H	H⋯*A*	*D*⋯*A*	*D*—H⋯*A*
O1—H1⋯O2	0.84	2.22	2.685 (3)	115
O1—H1⋯O9^i^	0.84	1.93	2.693 (3)	150
O1*A*—H1*A*⋯O8^ii^	0.84	2.31	2.978 (17)	136
O2—H2⋯O1*A*	0.84	2.29	2.693 (16)	110
O2—H2⋯O7^iii^	0.84	2.60	3.082 (3)	117
O2—H2⋯O11^ii^	0.84	2.13	2.952 (6)	166
O9—H9*A*⋯O3^iv^	0.8323 (17)	1.9961 (16)	2.821 (2)	170.86 (13)
O9—H9*B*⋯O10^iv^	0.8033 (17)	2.19 (6)	2.96 (6)	160.0 (18)
O9—H9*B*⋯O10	0.8033 (17)	1.98 (6)	2.73 (6)	155.0 (16)
O9—H9*B*⋯O10*A* ^iv^	0.8033 (17)	2.02 (4)	2.80 (4)	164.8 (14)
O9—H9*B*⋯O10*A*	0.8033 (17)	2.08 (4)	2.83 (4)	156.2 (12)
O11—H11*A*⋯O4^v^	0.840 (2)	2.1244 (16)	2.957 (3)	171.19 (17)
O11—H11*B*⋯O8^vi^	0.829 (3)	2.0583 (19)	2.873 (3)	167.4 (4)
O11*A*—H11*C*⋯O6^vi^	0.843 (8)	2.5753 (18)	3.290 (8)	143.3 (5)
O11*A*—H11*C*⋯O8^vi^	0.843 (8)	1.9964 (18)	2.776 (8)	153.2 (8)
O11*A*—H11*D*⋯O1	0.862 (18)	2.1019 (19)	2.851 (17)	145.0 (5)
O10—H10*A*⋯O2^iii^	0.81 (6)	2.2562 (16)	2.93 (6)	141 (4)
O10—H10*B*⋯O6	1.02 (6)	2.2175 (18)	3.14 (6)	150 (3)
O10*A*—H10*C*⋯O2^vii^	0.90 (5)	2.3694 (16)	3.13 (5)	142 (3)
O10*A*—H10*D*⋯O6	0.86 (4)	2.3000 (17)	3.10 (5)	155 (3)

Symmetry codes: (i) 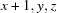; (ii) 
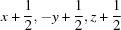
; (iii) 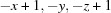; (iv) 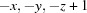; (v) 
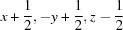
; (vi) 
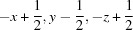
; (vii) 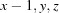.

**Table 2:** Hydrogen-bond geometry (Å, °) for Na_2_(tiron)·H_2_O[Chem scheme1]

*D*—H⋯*A*	*D*—H	H⋯*A*	*D*⋯*A*	*D*—H⋯*A*
O1—H1⋯O3^i^	0.82 (2)	2.05 (2)	2.8256 (15)	156 (2)
O2—H2⋯O4^ii^	0.84	1.98	2.8145 (14)	169
O9—H9*A*⋯O2^ii^	0.82 (3)	2.18 (3)	2.9904 (15)	173 (3)
O9—H9*B*⋯O7^iii^	0.80 (3)	2.14 (3)	2.8975 (15)	158 (3)

Symmetry codes: (i) 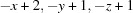; (ii) 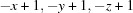; (iii) 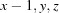.

**Table 3:** Hydrogen-bond geometry (Å, °) for (NH_4_)_2_(tiron)·H_2_O[Chem scheme1]

*D*—H⋯*A*	*D*—H	H⋯*A*	*D*⋯*A*	*D*—H⋯*A*
O1—H1⋯O5^i^	0.84	2.47	2.974 (4)	119
O1—H1⋯O8	0.84	2.06	2.790 (4)	146
O2—H2⋯O9^ii^	0.84	1.99	2.830 (5)	174
O9—H9*A*⋯O3^iii^	0.85 (2)	2.03 (2)	2.871 (5)	171 (5)
O9—H9*B*⋯O4	0.86 (2)	1.97 (2)	2.831 (4)	174 (5)
N1—H1*A*⋯O4^iv^	0.95 (2)	2.13 (3)	2.980 (5)	148 (3)
N1—H1*A*⋯O8^iii^	0.95 (2)	2.30 (4)	2.907 (5)	121 (3)
N1—H1*B*⋯O5^v^	0.95 (2)	2.11 (2)	2.996 (5)	155 (3)
N1—H1*C*⋯O1^vi^	0.95 (2)	2.03 (3)	2.850 (5)	143 (3)
N1—H1*C*⋯O2^vi^	0.95 (2)	2.63 (3)	3.470 (5)	147 (3)
N1—H1*D*⋯O3^i^	0.96 (2)	2.41 (4)	2.891 (5)	111 (3)
N1—H1*D*⋯O7	0.96 (2)	2.02 (3)	2.847 (5)	143 (3)
N2—H2*A*⋯O9	0.97 (2)	1.95 (2)	2.917 (5)	174 (3)
N2—H2*B*⋯O7	0.97 (2)	1.87 (2)	2.834 (5)	171 (3)
N2—H2*C*⋯O6^iii^	0.95 (2)	2.03 (3)	2.901 (5)	152 (3)
N2—H2*C*⋯O7^vii^	0.95 (2)	2.60 (3)	3.215 (5)	123 (3)
N2—H2*D*⋯O3^viii^	0.96 (2)	2.45 (4)	3.025 (5)	119 (3)
N2—H2*D*⋯O4^viii^	0.96 (2)	1.99 (2)	2.916 (5)	161 (3)
N2—H2*D*⋯O8^vii^	0.96 (2)	2.60 (4)	3.113 (5)	114 (3)

Symmetry codes: (i) 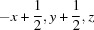; (ii) 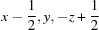; (iii) 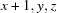; (iv) 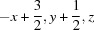; (v) 
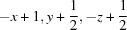
; (vi) 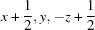; (vii) 
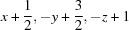
; (viii) 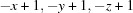.

**Table 4:** H-bonding to all NH_4_^+^ based protons in (NH_4_)_2_(tiron)·H_2_O

Proton on NH_4_	Acceptor/moiety	H-bond distance (Å)
N_1_H_1*a*_	O4/sulfonate	2.13 (3)
N_1_H_1*b*_	O5/sulfonate	2.10 (2)
N_1_H_1*c*_	O1/phenolic	2.04 (3)
N_1_H_1*d*_	O7/sulfonate	2.02 (3)
N_2_H_2*a*_	O9/water	1.95 (2)
N_2_H_2*b*_	O7/sulfonate	1.87 (2)
N_2_H_2*c*_	O6/sulfonate	2.03 (3)
N_2_H_2*d*_	O4/sulfonate	1.99 (2)

**Table 5:** Crystallographically characterized tiron salts

CSD code	Counter-cation	Observed coordination number	Charge of tiron	Inter­centroid distance (Å)	Reference
CAZZEI	Na^+^	2, 1, 1	3^−^	3.857	(Riley *et al.*, 1983 ▸)
HUCMOH	Ba^2+^	9	2^−^	3.520	(Côté & Shimizu, 2003 ▸)
OMARAV	Ca^2+^	8	2^−^	3.598	(Côté & Shimizu, 2003 ▸)
OMAREZ	Sr^2+^	9	2^−^	3.654	(Côté & Shimizu, 2003 ▸)
OMARID	Mg^2+^	6 (all water)	2^−^	4.180	(Côté & Shimizu, 2003 ▸)
FIMBEJ	Zn^2+^	6 (no tiron)	2^−^	N/A	(Wang *et al.*, 2005 ▸)
NIWKUA	Cd^2+^	6 (one sulfonate)	2^−^	N/A	(Zhang *et al.*, 2008 ▸)
FIRMEA	Cu^2+^	6 (one sulfonate)	2^−^	N/A	(Lu *et al.*, 2014 ▸)
TUYNUY	Mg^2+^	6 (no tiron)	2^−^	N/A	(Guan, 2016 ▸)
ADOXUP	La^3+^	9	3^−^	3.530	(Guan & Wang, 2016 ▸)
HUCMOH02	Ba^2+^	9	2^−^	3.516	(Guan & Wang, 2016 ▸)

**Table 6:** Experimental details

	Li_2_(tiron)·2.5H_2_O	Na_2_(tiron)·H_2_	(NH_4_)_2_(tiron)·H_2_O
Crystal data			
Chemical formula	[Li_2_(C_6_H_4_O_8_S_2_)(H_2_O)_2_]_2_·H_2_O	2Na^+^·C_6_H_6_O_9_S_2_^2−^	2NH_4_^+^·C_6_H_4_O_8_S_2_^2−^·H_2_O
*M*_r_	654.26	332.21	322.31
Crystal system, space group	Monoclinic, *P*2_1_/*n*	Monoclinic, *P*2_1_/*n*	Orthorhombic, *P* *b* *c* *a*
Temperature (K)	100	100	100
*a*, *b*, *c* (Å)	9.5847 (18), 7.4498 (15), 17.599 (4)	6.8156 (7), 16.1449 (15), 9.5870 (9)	6.5023 (15), 18.779 (4), 20.236 (4)
α, β, γ (°)	90, 102.997 (4), 90	90, 92.727 (2), 90	90, 90, 90
*V* (Å^3^)	1224.5 (4)	1053.73 (18)	2470.9 (9)
*Z*	2	4	8
Radiation type	Mo *K*α	Mo *K*α	Mo *K*α
μ (mm^−1^)	0.49	0.63	0.48
Crystal size (mm)	0.24 × 0.09 × 0.04	0.45 × 0.16 × 0.13	0.10 × 0.05 × 0.04

Data collection			
Diffractometer	Bruker APEXII	Bruker APEXII	Bruker APEXII
Absorption correction	Multi-scan (*SADABS*; Bruker, 2014 ▸)	Multi-scan (*SADABS*; Bruker, 2014 ▸)	Multi-scan (*SADABS*; Bruker, 2014 ▸)
*T*_min_, *T*_max_	0.670, 0.746	0.676, 0.746	0.663, 0.746
No. of measured, independent and observed [*I* > 2σ(*I*)] reflections	15864, 2851, 2313	9573, 2485, 2340	12544, 2255, 1354
*R*_int_	0.049	0.025	0.147

Refinement			
*R*[*F* ^2^ > 2σ(*F* ^2^)], *wR*(*F* ^2^), *S*	0.041, 0.100, 1.02	0.023, 0.071, 1.08	0.055, 0.127, 1.01
No. of reflections	2851	2485	2255
No. of parameters	217	185	204
No. of restraints	18	0	23
H-atom treatment	H-atom parameters constrained	H atoms treated by a mixture of independent and constrained refinement	H atoms treated by a mixture of independent and constrained refinement
Δρ_max_, Δρ_min_ (e Å^−3^)	0.44, −0.48	0.44, −0.43	0.42, −0.53

Computer programs: *APEX2* and *SAINT* (Bruker, 2014 ▸), *SAINT* (Bruker, 2016 ▸), *SHELXS97* (Sheldrick, 2008 ▸), *SHELXL* (Sheldrick, 2015 ▸) and *OLEX2* (Dolomanov *et al.*, 2009 ▸).

## supplementary crystallographic information

### Disodium 4,5-dihydroxybenzene-1,3-disulfonate (NaTiron) Crystal data

**Table:**

2Na^+^·C_6_H_6_O_9_S_2_^2^^−^	*F*(000) = 672
*M**_r_* = 332.21	*D*_x_ = 2.094 Mg m^−^^3^
Monoclinic, *P*2_1_/*n*	Mo *K*α radiation, λ = 0.71073 Å
*a* = 6.8156 (7) Å	Cell parameters from 6900 reflections
*b* = 16.1449 (15) Å	θ = 2.5–27.9°
*c* = 9.5870 (9) Å	µ = 0.63 mm^−^^1^
β = 92.727 (2)°	*T* = 100 K
*V* = 1053.73 (18) Å^3^	Plank, colorless
*Z* = 4	0.45 × 0.16 × 0.13 mm

### Disodium 4,5-dihydroxybenzene-1,3-disulfonate (NaTiron) Data collection

**Table:**

Bruker APEXII diffractometer	2485 independent reflections
Radiation source: sealed tube	2340 reflections with *I* > 2σ(*I*)
Graphite monochromator	*R*_int_ = 0.025
Detector resolution: 8.333 pixels mm^-1^	θ_max_ = 27.9°, θ_min_ = 2.5°
ω and φ scans	*h* = −8→8
Absorption correction: multi-scan (SADABS; Bruker, 2014)	*k* = −17→21
*T*_min_ = 0.676, *T*_max_ = 0.746	*l* = −11→12
9573 measured reflections	

### Disodium 4,5-dihydroxybenzene-1,3-disulfonate (NaTiron) Refinement

**Table:**

Refinement on *F*^2^	Primary atom site location: dual
Least-squares matrix: full	Hydrogen site location: mixed
*R*[*F*^2^ > 2σ(*F*^2^)] = 0.023	H atoms treated by a mixture of independent and constrained refinement
*wR*(*F*^2^) = 0.071	*w* = 1/[σ^2^(*F*_o_^2^) + (0.0406*P*)^2^ + 0.6133*P*] where *P* = (*F*_o_^2^ + 2*F*_c_^2^)/3
*S* = 1.08	(Δ/σ)_max_ = 0.001
2485 reflections	Δρ_max_ = 0.44 e Å^−^^3^
185 parameters	Δρ_min_ = −0.43 e Å^−^^3^
0 restraints	

### Disodium 4,5-dihydroxybenzene-1,3-disulfonate (NaTiron) Special details

**Table:**

Geometry. All esds (except the esd in the dihedral angle between two l.s. planes) are estimated using the full covariance matrix. The cell esds are taken into account individually in the estimation of esds in distances, angles and torsion angles; correlations between esds in cell parameters are only used when they are defined by crystal symmetry. An approximate (isotropic) treatment of cell esds is used for estimating esds involving l.s. planes.

### Disodium 4,5-dihydroxybenzene-1,3-disulfonate (NaTiron) Fractional atomic coordinates and isotropic or equivalent isotropic displacement parameters (Å^2^)

**Table:**

	*x*	*y*	*z*	*U*_iso_*/*U*_eq_	
S1	0.74934 (5)	0.47581 (2)	0.82422 (3)	0.00662 (9)	
S2	0.78290 (5)	0.28628 (2)	0.35605 (3)	0.00666 (9)	
Na1	0.52212 (8)	0.15609 (3)	0.55730 (6)	0.01024 (13)	
Na2	0.52978 (8)	0.35866 (3)	0.07130 (6)	0.00904 (13)	
O1	0.77862 (15)	0.44913 (6)	0.21044 (10)	0.0095 (2)	
H1	0.834 (3)	0.4913 (15)	0.184 (2)	0.026 (6)*	
O2	0.72832 (15)	0.60443 (6)	0.33613 (10)	0.0103 (2)	
H2	0.646843	0.597179	0.268721	0.015*	
O3	0.93352 (15)	0.43988 (6)	0.88058 (10)	0.0102 (2)	
O4	0.57824 (15)	0.42796 (6)	0.86349 (10)	0.0104 (2)	
O5	0.73253 (14)	0.56359 (6)	0.85674 (10)	0.0094 (2)	
O6	0.78663 (15)	0.22645 (6)	0.47032 (10)	0.0089 (2)	
O7	0.96390 (15)	0.28408 (6)	0.27946 (10)	0.0104 (2)	
O8	0.60507 (15)	0.28136 (6)	0.26724 (10)	0.0102 (2)	
O9	0.26274 (16)	0.24089 (7)	0.49210 (11)	0.0115 (2)	
H9A	0.256 (4)	0.2814 (17)	0.542 (3)	0.042 (7)*	
H9B	0.205 (4)	0.2515 (16)	0.419 (3)	0.037 (7)*	
C1	0.76633 (19)	0.45634 (8)	0.35261 (13)	0.0074 (2)	
C2	0.74189 (19)	0.53371 (8)	0.41504 (14)	0.0078 (3)	
C3	0.73789 (19)	0.53988 (8)	0.55936 (14)	0.0082 (2)	
H3	0.723889	0.592459	0.602256	0.010*	
C4	0.75445 (19)	0.46884 (8)	0.64062 (13)	0.0073 (2)	
C5	0.77038 (19)	0.39085 (8)	0.58031 (13)	0.0082 (3)	
H5	0.778109	0.342517	0.636828	0.010*	
C6	0.77476 (19)	0.38501 (8)	0.43624 (14)	0.0072 (2)	

### Disodium 4,5-dihydroxybenzene-1,3-disulfonate (NaTiron) Atomic displacement parameters (Å^2^)

**Table:**

	*U*^11^	*U*^22^	*U*^33^	*U*^12^	*U*^13^	*U*^23^
S1	0.00802 (17)	0.00652 (17)	0.00529 (15)	−0.00008 (11)	−0.00004 (11)	−0.00033 (10)
S2	0.00871 (17)	0.00549 (16)	0.00574 (16)	0.00048 (11)	−0.00007 (12)	0.00022 (11)
Na1	0.0092 (3)	0.0105 (3)	0.0109 (3)	0.0003 (2)	−0.0001 (2)	−0.0014 (2)
Na2	0.0093 (3)	0.0099 (3)	0.0079 (3)	0.0004 (2)	−0.0002 (2)	0.0004 (2)
O1	0.0137 (5)	0.0090 (5)	0.0060 (4)	−0.0021 (4)	0.0006 (3)	0.0014 (4)
O2	0.0152 (5)	0.0072 (5)	0.0081 (4)	−0.0012 (4)	−0.0029 (4)	0.0030 (4)
O3	0.0105 (5)	0.0108 (5)	0.0091 (4)	0.0022 (4)	−0.0018 (3)	0.0007 (4)
O4	0.0111 (5)	0.0121 (5)	0.0079 (4)	−0.0035 (4)	0.0009 (3)	0.0017 (4)
O5	0.0110 (5)	0.0071 (5)	0.0100 (4)	0.0005 (4)	0.0007 (3)	−0.0018 (3)
O6	0.0113 (5)	0.0073 (4)	0.0081 (4)	0.0008 (4)	0.0003 (4)	0.0024 (3)
O7	0.0116 (5)	0.0111 (5)	0.0087 (5)	0.0022 (4)	0.0031 (4)	0.0003 (3)
O8	0.0118 (5)	0.0095 (5)	0.0091 (5)	−0.0010 (4)	−0.0029 (4)	0.0005 (3)
O9	0.0135 (5)	0.0114 (5)	0.0095 (5)	0.0012 (4)	−0.0007 (4)	−0.0003 (4)
C1	0.0066 (6)	0.0090 (6)	0.0065 (6)	−0.0009 (5)	0.0000 (4)	0.0007 (5)
C2	0.0072 (6)	0.0069 (6)	0.0093 (6)	−0.0006 (5)	−0.0003 (5)	0.0021 (5)
C3	0.0073 (6)	0.0074 (6)	0.0100 (6)	−0.0008 (5)	0.0003 (5)	0.0000 (5)
C4	0.0068 (6)	0.0091 (6)	0.0059 (6)	−0.0007 (5)	−0.0001 (4)	0.0003 (5)
C5	0.0079 (6)	0.0080 (6)	0.0086 (6)	−0.0002 (5)	0.0004 (5)	0.0013 (5)
C6	0.0065 (6)	0.0064 (6)	0.0087 (6)	0.0004 (5)	0.0002 (4)	−0.0007 (5)

### Disodium 4,5-dihydroxybenzene-1,3-disulfonate (NaTiron) Geometric parameters (Å, º)

**Table:**

S1—O3	1.4628 (10)	Na2—O5^vi^	2.3163 (11)
S1—O4	1.4630 (10)	Na2—O6^iii^	2.3278 (11)
S1—O5	1.4567 (10)	Na2—O8	2.2929 (11)
S1—C4	1.7657 (13)	Na2—O9^vii^	2.4073 (12)
S2—Na2	3.3683 (7)	O1—H1	0.82 (2)
S2—O6	1.4599 (10)	O1—C1	1.3746 (16)
S2—O7	1.4658 (10)	O2—H2	0.8400
S2—O8	1.4499 (10)	O2—C2	1.3704 (16)
S2—C6	1.7717 (14)	O9—H9A	0.82 (3)
Na1—Na2^i^	3.3731 (8)	O9—H9B	0.80 (3)
Na1—Na2^ii^	3.4644 (8)	C1—C2	1.3984 (18)
Na1—O1^i^	2.8332 (12)	C1—C6	1.4028 (18)
Na1—O3^iii^	2.3534 (11)	C2—C3	1.3888 (19)
Na1—O5^iv^	2.3611 (11)	C3—H3	0.9500
Na1—O6	2.3192 (12)	C3—C4	1.3881 (18)
Na1—O7^i^	2.3890 (11)	C4—C5	1.3920 (19)
Na1—O9	2.2988 (12)	C5—H5	0.9500
Na2—O1	2.5640 (12)	C5—C6	1.3862 (18)
Na2—O4^v^	2.3223 (11)		
			
O3—S1—O4	112.09 (6)	O5^vi^—Na2—O9^vii^	170.31 (4)
O3—S1—C4	106.60 (6)	O6^iii^—Na2—O1	173.13 (4)
O4—S1—C4	106.06 (6)	O6^iii^—Na2—O9^vii^	86.67 (4)
O5—S1—O3	112.43 (6)	O8—Na2—O1	76.54 (4)
O5—S1—O4	112.76 (6)	O8—Na2—O4^v^	158.38 (4)
O5—S1—C4	106.31 (6)	O8—Na2—O5^vi^	101.35 (4)
O6—S2—Na2	145.86 (4)	O8—Na2—O6^iii^	98.43 (4)
O6—S2—O7	112.01 (6)	O8—Na2—O9^vii^	76.69 (4)
O6—S2—C6	105.63 (6)	O9^vii^—Na2—O1	96.58 (4)
O7—S2—Na2	90.76 (4)	Na1^vii^—O1—H1	92.7 (16)
O7—S2—C6	106.48 (6)	Na2—O1—Na1^vii^	77.18 (3)
O8—S2—Na2	33.02 (4)	Na2—O1—H1	127.7 (15)
O8—S2—O6	112.87 (6)	C1—O1—Na1^vii^	129.01 (8)
O8—S2—O7	113.86 (6)	C1—O1—Na2	119.61 (8)
O8—S2—C6	105.17 (6)	C1—O1—H1	106.6 (15)
C6—S2—Na2	90.81 (5)	C2—O2—H2	109.5
Na2^i^—Na1—Na2^ii^	170.83 (3)	S1—O3—Na1^ii^	135.50 (6)
O1^i^—Na1—Na2^i^	47.83 (2)	S1—O4—Na2^viii^	128.53 (6)
O1^i^—Na1—Na2^ii^	123.18 (3)	S1—O5—Na1^ix^	128.93 (6)
O3^iii^—Na1—Na2^ii^	101.78 (3)	S1—O5—Na2^vi^	131.35 (6)
O3^iii^—Na1—Na2^i^	76.14 (3)	Na2^vi^—O5—Na1^ix^	95.57 (4)
O3^iii^—Na1—O1^i^	80.89 (4)	S2—O6—Na1	127.43 (6)
O3^iii^—Na1—O5^iv^	89.31 (4)	S2—O6—Na2^ii^	133.57 (6)
O3^iii^—Na1—O7^i^	149.34 (4)	Na1—O6—Na2^ii^	96.41 (4)
O5^iv^—Na1—Na2^i^	129.11 (3)	S2—O7—Na1^vii^	128.04 (6)
O5^iv^—Na1—Na2^ii^	41.72 (3)	S2—O8—Na2	126.83 (6)
O5^iv^—Na1—O1^i^	82.09 (4)	Na1—O9—Na2^i^	91.54 (4)
O5^iv^—Na1—O7^i^	95.13 (4)	Na1—O9—H9A	112.4 (19)
O6—Na1—Na2^ii^	41.89 (3)	Na1—O9—H9B	134.7 (18)
O6—Na1—Na2^i^	147.21 (3)	Na2^i^—O9—H9A	107.1 (19)
O6—Na1—O1^i^	164.68 (4)	Na2^i^—O9—H9B	96.3 (19)
O6—Na1—O3^iii^	103.94 (4)	H9A—O9—H9B	108 (2)
O6—Na1—O5^iv^	83.43 (4)	O1—C1—C2	120.94 (12)
O6—Na1—O7^i^	106.70 (4)	O1—C1—C6	119.63 (12)
O7^i^—Na1—Na2^ii^	101.61 (3)	C2—C1—C6	119.42 (12)
O7^i^—Na1—Na2^i^	77.60 (3)	O2—C2—C1	120.95 (12)
O7^i^—Na1—O1^i^	69.77 (3)	O2—C2—C3	119.09 (12)
O9—Na1—Na2^ii^	143.62 (4)	C3—C2—C1	119.93 (12)
O9—Na1—Na2^i^	45.51 (3)	C2—C3—H3	120.2
O9—Na1—O1^i^	92.07 (4)	C4—C3—C2	119.66 (12)
O9—Na1—O3^iii^	91.64 (4)	C4—C3—H3	120.2
O9—Na1—O5^iv^	173.87 (4)	C3—C4—S1	120.12 (10)
O9—Na1—O6	102.21 (4)	C3—C4—C5	121.34 (12)
O9—Na1—O7^i^	80.99 (4)	C5—C4—S1	118.52 (10)
O4^v^—Na2—O1	93.11 (4)	C4—C5—H5	120.6
O4^v^—Na2—O6^iii^	93.16 (4)	C6—C5—C4	118.76 (12)
O4^v^—Na2—O9^vii^	85.88 (4)	C6—C5—H5	120.6
O5^vi^—Na2—O1	92.13 (4)	C1—C6—S2	119.47 (10)
O5^vi^—Na2—O4^v^	97.91 (4)	C5—C6—S2	119.74 (10)
O5^vi^—Na2—O6^iii^	84.23 (4)	C5—C6—C1	120.76 (12)
			
S1—C4—C5—C6	−179.90 (10)	O6—S2—C6—C1	−178.85 (11)
Na1^vii^—O1—C1—C2	141.05 (10)	O6—S2—C6—C5	−0.93 (12)
Na1^vii^—O1—C1—C6	−40.13 (17)	O7—S2—O6—Na1	−148.47 (7)
Na2—S2—O6—Na1	−20.03 (12)	O7—S2—O6—Na2^ii^	8.76 (10)
Na2—S2—O6—Na2^ii^	137.20 (6)	O7—S2—O8—Na2	−49.16 (9)
Na2—S2—O7—Na1^vii^	11.89 (7)	O7—S2—C6—C1	61.90 (12)
Na2—S2—C6—C1	−29.14 (11)	O7—S2—C6—C5	−120.18 (11)
Na2—S2—C6—C5	148.78 (11)	O8—S2—O6—Na1	−18.39 (9)
Na2—O1—C1—C2	−121.37 (12)	O8—S2—O6—Na2^ii^	138.85 (8)
Na2—O1—C1—C6	57.45 (15)	O8—S2—O7—Na1^vii^	36.24 (9)
O1—C1—C2—O2	0.4 (2)	O8—S2—C6—C1	−59.25 (12)
O1—C1—C2—C3	−177.37 (12)	O8—S2—C6—C5	118.67 (11)
O1—C1—C6—S2	−4.63 (17)	C1—C2—C3—C4	−1.2 (2)
O1—C1—C6—C5	177.47 (12)	C2—C1—C6—S2	174.21 (10)
O2—C2—C3—C4	−179.04 (12)	C2—C1—C6—C5	−3.7 (2)
O3—S1—O4—Na2^viii^	35.85 (9)	C2—C3—C4—S1	−179.94 (10)
O3—S1—O5—Na1^ix^	13.55 (9)	C2—C3—C4—C5	−1.6 (2)
O3—S1—O5—Na2^vi^	−137.77 (7)	C3—C4—C5—C6	1.7 (2)
O3—S1—C4—C3	−121.82 (11)	C4—S1—O3—Na1^ii^	−132.68 (8)
O3—S1—C4—C5	59.75 (12)	C4—S1—O4—Na2^viii^	151.80 (7)
O4—S1—O3—Na1^ii^	−17.06 (10)	C4—S1—O5—Na1^ix^	−102.73 (8)
O4—S1—O5—Na1^ix^	141.46 (7)	C4—S1—O5—Na2^vi^	105.95 (8)
O4—S1—O5—Na2^vi^	−9.86 (10)	C4—C5—C6—S2	−176.94 (10)
O4—S1—C4—C3	118.57 (11)	C4—C5—C6—C1	1.0 (2)
O4—S1—C4—C5	−59.86 (12)	C6—S2—O6—Na1	96.00 (8)
O5—S1—O3—Na1^ii^	111.21 (9)	C6—S2—O6—Na2^ii^	−106.76 (8)
O5—S1—O4—Na2^viii^	−92.24 (8)	C6—S2—O7—Na1^vii^	−79.18 (8)
O5—S1—C4—C3	−1.68 (13)	C6—S2—O8—Na2	67.03 (8)
O5—S1—C4—C5	179.89 (10)	C6—C1—C2—O2	−178.42 (12)
O6—S2—O7—Na1^vii^	165.81 (6)	C6—C1—C2—C3	3.8 (2)
O6—S2—O8—Na2	−178.30 (6)		

Symmetry codes: (i) *x*−1/2, −*y*+1/2, *z*+1/2; (ii) *x*+1/2, −*y*+1/2, *z*+1/2; (iii) *x*−1/2, −*y*+1/2, *z*−1/2; (iv) −*x*+3/2, *y*−1/2, −*z*+3/2; (v) *x*, *y*, *z*−1; (vi) −*x*+1, −*y*+1, −*z*+1; (vii) *x*+1/2, −*y*+1/2, *z*−1/2; (viii) *x*, *y*, *z*+1; (ix) −*x*+3/2, *y*+1/2, −*z*+3/2.

### Disodium 4,5-dihydroxybenzene-1,3-disulfonate (NaTiron) Hydrogen-bond geometry (Å, º)

**Table:**

*D*—H···*A*	*D*—H	H···*A*	*D*···*A*	*D*—H···*A*
O1—H1···O3^x^	0.82 (2)	2.05 (2)	2.8256 (15)	156 (2)
O2—H2···O4^vi^	0.84	1.98	2.8145 (14)	169
O9—H9*A*···O2^vi^	0.82 (3)	2.18 (3)	2.9904 (15)	173 (3)
O9—H9*B*···O7^xi^	0.80 (3)	2.14 (3)	2.8975 (15)	158 (3)

Symmetry codes: (vi) −*x*+1, −*y*+1, −*z*+1; (x) −*x*+2, −*y*+1, −*z*+1; (xi) *x*−1, *y*, *z*.

### Diammonium 4,5-dihydroxybenzene-1,3-disulfonate monohydrate (NH4Tiron) Crystal data

**Table:**

2NH_4_^+^·C_6_H_4_O_8_S_2_^2^^−^·H_2_O	*D*_x_ = 1.733 Mg m^−^^3^
*M**_r_* = 322.31	Mo *K*α radiation, λ = 0.71073 Å
Orthorhombic, *P**b**c**a*	Cell parameters from 511 reflections
*a* = 6.5023 (15) Å	θ = 3.0–21.0°
*b* = 18.779 (4) Å	µ = 0.48 mm^−^^1^
*c* = 20.236 (4) Å	*T* = 100 K
*V* = 2470.9 (9) Å^3^	Plank, colorless
*Z* = 8	0.10 × 0.05 × 0.04 mm
*F*(000) = 1344	

### Diammonium 4,5-dihydroxybenzene-1,3-disulfonate monohydrate (NH4Tiron) Data collection

**Table:**

Bruker APEXII diffractometer	2255 independent reflections
Radiation source: sealed tube	1354 reflections with *I* > 2σ(*I*)
Graphite monochromator	*R*_int_ = 0.147
Detector resolution: 8.333 pixels mm^-1^	θ_max_ = 25.3°, θ_min_ = 2.0°
ω and φ scans	*h* = −7→7
Absorption correction: multi-scan (SADABS; Bruker, 2014)	*k* = −22→22
*T*_min_ = 0.663, *T*_max_ = 0.746	*l* = −15→24
12544 measured reflections	

### Diammonium 4,5-dihydroxybenzene-1,3-disulfonate monohydrate (NH4Tiron) Refinement

**Table:**

Refinement on *F*^2^	23 restraints
Least-squares matrix: full	Hydrogen site location: mixed
*R*[*F*^2^ > 2σ(*F*^2^)] = 0.055	H atoms treated by a mixture of independent and constrained refinement
*wR*(*F*^2^) = 0.127	*w* = 1/[σ^2^(*F*_o_^2^) + (0.0411*P*)^2^ + 0.6346*P*] where *P* = (*F*_o_^2^ + 2*F*_c_^2^)/3
*S* = 1.01	(Δ/σ)_max_ < 0.001
2255 reflections	Δρ_max_ = 0.42 e Å^−^^3^
204 parameters	Δρ_min_ = −0.53 e Å^−^^3^

### Diammonium 4,5-dihydroxybenzene-1,3-disulfonate monohydrate (NH4Tiron) Special details

**Table:**

Geometry. All esds (except the esd in the dihedral angle between two l.s. planes) are estimated using the full covariance matrix. The cell esds are taken into account individually in the estimation of esds in distances, angles and torsion angles; correlations between esds in cell parameters are only used when they are defined by crystal symmetry. An approximate (isotropic) treatment of cell esds is used for estimating esds involving l.s. planes.

### Diammonium 4,5-dihydroxybenzene-1,3-disulfonate monohydrate (NH4Tiron) Fractional atomic coordinates and isotropic or equivalent isotropic displacement parameters (Å^2^)

**Table:**

	*x*	*y*	*z*	*U*_iso_*/*U*_eq_	
S1	0.29066 (17)	0.42273 (6)	0.34618 (5)	0.0132 (3)	
S2	0.26230 (17)	0.69952 (6)	0.42150 (6)	0.0154 (3)	
O1	0.2580 (5)	0.72630 (16)	0.27192 (15)	0.0216 (8)	
H1	0.224751	0.751861	0.304271	0.032*	
O2	0.2771 (5)	0.62901 (17)	0.17777 (13)	0.0175 (7)	
H2	0.270116	0.594058	0.151957	0.026*	
O3	0.0895 (5)	0.41015 (16)	0.37651 (14)	0.0184 (8)	
O4	0.4555 (5)	0.41571 (16)	0.39574 (14)	0.0152 (7)	
O5	0.3278 (5)	0.38099 (17)	0.28738 (14)	0.0199 (8)	
O6	0.2038 (5)	0.66240 (16)	0.48126 (15)	0.0193 (8)	
O7	0.4689 (5)	0.72999 (17)	0.42551 (15)	0.0209 (8)	
O8	0.1134 (5)	0.75262 (17)	0.39943 (16)	0.0234 (8)	
C1	0.2691 (7)	0.6571 (2)	0.2915 (2)	0.0127 (10)	
C2	0.2800 (7)	0.6052 (3)	0.2418 (2)	0.0152 (11)	
C3	0.2921 (7)	0.5343 (2)	0.2580 (2)	0.0129 (10)	
H3	0.301928	0.499446	0.224165	0.015*	
C4	0.2901 (7)	0.5134 (2)	0.3242 (2)	0.0119 (10)	
C5	0.2797 (7)	0.5640 (2)	0.3741 (2)	0.0138 (10)	
H5	0.279726	0.549780	0.419125	0.017*	
C6	0.2694 (6)	0.6352 (2)	0.3576 (2)	0.0115 (9)	
O9	0.7831 (5)	0.51345 (17)	0.41197 (17)	0.0209 (8)	
H9A	0.883 (5)	0.487 (2)	0.401 (2)	0.031*	
H9B	0.684 (5)	0.483 (2)	0.410 (2)	0.031*	
N1	0.7375 (6)	0.8117 (2)	0.34433 (18)	0.0172 (9)	
H1A	0.853 (4)	0.829 (2)	0.3686 (17)	0.026*	
H1B	0.707 (6)	0.8458 (17)	0.3109 (15)	0.026*	
H1C	0.762 (6)	0.7691 (14)	0.3204 (17)	0.026*	
H1D	0.620 (4)	0.804 (2)	0.3718 (16)	0.026*	
N2	0.7621 (6)	0.6433 (2)	0.49080 (17)	0.0159 (9)	
H2A	0.767 (6)	0.5981 (13)	0.4675 (17)	0.024*	
H2B	0.673 (5)	0.6738 (18)	0.4648 (16)	0.024*	
H2C	0.895 (4)	0.664 (2)	0.4954 (19)	0.024*	
H2D	0.698 (6)	0.634 (2)	0.5323 (12)	0.024*	

### Diammonium 4,5-dihydroxybenzene-1,3-disulfonate monohydrate (NH4Tiron) Atomic displacement parameters (Å^2^)

**Table:**

	*U*^11^	*U*^22^	*U*^33^	*U*^12^	*U*^13^	*U*^23^
S1	0.0118 (5)	0.0115 (6)	0.0162 (6)	0.0002 (5)	−0.0002 (5)	−0.0014 (5)
S2	0.0156 (6)	0.0120 (6)	0.0186 (6)	0.0004 (5)	−0.0001 (5)	−0.0024 (5)
O1	0.028 (2)	0.0126 (18)	0.0244 (18)	0.0027 (16)	0.0000 (17)	−0.0008 (14)
O2	0.0188 (18)	0.0202 (19)	0.0135 (15)	0.0027 (16)	0.0001 (15)	0.0018 (14)
O3	0.0134 (18)	0.016 (2)	0.0258 (18)	−0.0042 (14)	0.0062 (14)	0.0024 (15)
O4	0.0136 (17)	0.0127 (19)	0.0194 (16)	0.0014 (14)	−0.0035 (13)	0.0004 (14)
O5	0.032 (2)	0.0115 (18)	0.0158 (17)	0.0028 (15)	−0.0010 (15)	−0.0042 (14)
O6	0.024 (2)	0.0156 (19)	0.0185 (17)	0.0012 (15)	0.0059 (15)	−0.0035 (14)
O7	0.0184 (18)	0.020 (2)	0.0239 (18)	−0.0051 (14)	0.0004 (15)	−0.0033 (15)
O8	0.022 (2)	0.019 (2)	0.0291 (18)	0.0111 (16)	−0.0067 (17)	0.0004 (16)
C1	0.006 (2)	0.011 (2)	0.021 (2)	0.0002 (19)	0.005 (2)	0.0040 (19)
C2	0.008 (2)	0.021 (3)	0.016 (2)	−0.002 (2)	−0.006 (2)	0.005 (2)
C3	0.011 (2)	0.013 (2)	0.014 (2)	0.002 (2)	0.0008 (19)	−0.005 (2)
C4	0.009 (2)	0.008 (3)	0.018 (2)	−0.0018 (19)	−0.002 (2)	0.0014 (19)
C5	0.008 (2)	0.017 (3)	0.017 (2)	−0.001 (2)	−0.0017 (19)	0.004 (2)
C6	0.006 (2)	0.011 (2)	0.017 (2)	−0.0003 (18)	−0.001 (2)	0.0023 (19)
O9	0.0136 (18)	0.020 (2)	0.0290 (19)	−0.0020 (15)	−0.0015 (16)	−0.0015 (16)
N1	0.014 (2)	0.018 (2)	0.019 (2)	−0.0015 (18)	−0.0003 (19)	−0.0002 (18)
N2	0.019 (2)	0.015 (2)	0.014 (2)	0.0006 (19)	0.0029 (19)	0.0010 (17)

### Diammonium 4,5-dihydroxybenzene-1,3-disulfonate monohydrate (NH4Tiron) Geometric parameters (Å, º)

**Table:**

S1—O3	1.464 (3)	C3—H3	0.9500
S1—O4	1.474 (3)	C3—C4	1.396 (6)
S1—O5	1.445 (3)	C4—C5	1.388 (6)
S1—C4	1.760 (4)	C5—H5	0.9500
S2—O6	1.447 (3)	C5—C6	1.378 (6)
S2—O7	1.462 (3)	O9—H9A	0.849 (19)
S2—O8	1.460 (3)	O9—H9B	0.861 (19)
S2—C6	1.770 (4)	N1—H1A	0.951 (18)
O1—H1	0.8400	N1—H1B	0.954 (17)
O1—C1	1.361 (5)	N1—H1C	0.949 (17)
O2—H2	0.8400	N1—H1D	0.956 (17)
O2—C2	1.370 (5)	N2—H2A	0.972 (17)
C1—C2	1.402 (6)	N2—H2B	0.969 (17)
C1—C6	1.401 (6)	N2—H2C	0.948 (17)
C2—C3	1.373 (6)	N2—H2D	0.955 (17)
			
O3—S1—O4	110.49 (18)	C3—C4—S1	121.0 (3)
O3—S1—C4	105.1 (2)	C5—C4—S1	118.6 (3)
O4—S1—C4	105.1 (2)	C5—C4—C3	120.4 (4)
O5—S1—O3	114.01 (19)	C4—C5—H5	120.3
O5—S1—O4	112.96 (19)	C6—C5—C4	119.4 (4)
O5—S1—C4	108.5 (2)	C6—C5—H5	120.3
O6—S2—O7	112.6 (2)	C1—C6—S2	119.8 (3)
O6—S2—O8	114.2 (2)	C5—C6—S2	119.1 (3)
O6—S2—C6	106.75 (19)	C5—C6—C1	121.0 (4)
O7—S2—C6	106.47 (19)	H9A—O9—H9B	100 (3)
O8—S2—O7	111.0 (2)	H1A—N1—H1B	108 (3)
O8—S2—C6	105.1 (2)	H1A—N1—H1C	114 (3)
C1—O1—H1	109.5	H1A—N1—H1D	112 (3)
C2—O2—H2	109.5	H1B—N1—H1C	104 (3)
O1—C1—C2	117.3 (4)	H1B—N1—H1D	110 (3)
O1—C1—C6	123.9 (4)	H1C—N1—H1D	108 (3)
C6—C1—C2	118.8 (4)	H2A—N2—H2B	106 (3)
O2—C2—C1	116.8 (4)	H2A—N2—H2C	112 (3)
O2—C2—C3	122.9 (4)	H2A—N2—H2D	106 (3)
C3—C2—C1	120.3 (4)	H2B—N2—H2C	111 (3)
C2—C3—H3	119.9	H2B—N2—H2D	109 (3)
C2—C3—C4	120.1 (4)	H2C—N2—H2D	113 (3)
C4—C3—H3	119.9		
			
S1—C4—C5—C6	−177.0 (3)	O7—S2—C6—C1	−76.0 (4)
O1—C1—C2—O2	−0.4 (6)	O7—S2—C6—C5	102.6 (4)
O1—C1—C2—C3	179.8 (4)	O8—S2—C6—C1	41.9 (4)
O1—C1—C6—S2	−1.8 (6)	O8—S2—C6—C5	−139.5 (4)
O1—C1—C6—C5	179.6 (4)	C1—C2—C3—C4	1.1 (6)
O2—C2—C3—C4	−178.6 (4)	C2—C1—C6—S2	178.4 (3)
O3—S1—C4—C3	−112.9 (4)	C2—C1—C6—C5	−0.2 (6)
O3—S1—C4—C5	64.7 (4)	C2—C3—C4—S1	176.4 (4)
O4—S1—C4—C3	130.5 (4)	C2—C3—C4—C5	−1.2 (7)
O4—S1—C4—C5	−51.9 (4)	C3—C4—C5—C6	0.6 (6)
O5—S1—C4—C3	9.4 (4)	C4—C5—C6—S2	−178.5 (3)
O5—S1—C4—C5	−173.0 (3)	C4—C5—C6—C1	0.1 (6)
O6—S2—C6—C1	163.6 (3)	C6—C1—C2—O2	179.3 (4)
O6—S2—C6—C5	−17.8 (4)	C6—C1—C2—C3	−0.4 (6)

### Diammonium 4,5-dihydroxybenzene-1,3-disulfonate monohydrate (NH4Tiron) Hydrogen-bond geometry (Å, º)

**Table:**

*D*—H···*A*	*D*—H	H···*A*	*D*···*A*	*D*—H···*A*
O1—H1···O5^i^	0.84	2.47	2.974 (4)	119
O1—H1···O8	0.84	2.06	2.790 (4)	146
O2—H2···O9^ii^	0.84	1.99	2.830 (5)	174
O9—H9*A*···O3^iii^	0.85 (2)	2.03 (2)	2.871 (5)	171 (5)
O9—H9*B*···O4	0.86 (2)	1.97 (2)	2.831 (4)	174 (5)
N1—H1*A*···O4^iv^	0.95 (2)	2.13 (3)	2.980 (5)	148 (3)
N1—H1*A*···O8^iii^	0.95 (2)	2.30 (4)	2.907 (5)	121 (3)
N1—H1*B*···O5^v^	0.95 (2)	2.11 (2)	2.996 (5)	155 (3)
N1—H1*C*···O1^vi^	0.95 (2)	2.03 (3)	2.850 (5)	143 (3)
N1—H1*C*···O2^vi^	0.95 (2)	2.63 (3)	3.470 (5)	147 (3)
N1—H1*D*···O3^i^	0.96 (2)	2.41 (4)	2.891 (5)	111 (3)
N1—H1*D*···O7	0.96 (2)	2.02 (3)	2.847 (5)	143 (3)
N2—H2*A*···O9	0.97 (2)	1.95 (2)	2.917 (5)	174 (3)
N2—H2*B*···O7	0.97 (2)	1.87 (2)	2.834 (5)	171 (3)
N2—H2*C*···O6^iii^	0.95 (2)	2.03 (3)	2.901 (5)	152 (3)
N2—H2*C*···O7^vii^	0.95 (2)	2.60 (3)	3.215 (5)	123 (3)
N2—H2*D*···O3^viii^	0.96 (2)	2.45 (4)	3.025 (5)	119 (3)
N2—H2*D*···O4^viii^	0.96 (2)	1.99 (2)	2.916 (5)	161 (3)
N2—H2*D*···O8^vii^	0.96 (2)	2.60 (4)	3.113 (5)	114 (3)

Symmetry codes: (i) −*x*+1/2, *y*+1/2, *z*; (ii) *x*−1/2, *y*, −*z*+1/2; (iii) *x*+1, *y*, *z*; (iv) −*x*+3/2, *y*+1/2, *z*; (v) −*x*+1, *y*+1/2, −*z*+1/2; (vi) *x*+1/2, *y*, −*z*+1/2; (vii) *x*+1/2, −*y*+3/2, −*z*+1; (viii) −*x*+1, −*y*+1, −*z*+1.

### µ-4,5-Dihydroxybenzene-1,3-disulfonato-bis[aqualithium(I)] hemihydrate (LiTiron) Crystal data

**Table:**

[Li_2_(C_6_H_4_O_8_S_2_)(H_2_O)_2_]_2_·H_2_O	*F*(000) = 668
*M**_r_* = 654.26	*D*_x_ = 1.775 Mg m^−^^3^
Monoclinic, *P*2_1_/*n*	Mo *K*α radiation, λ = 0.71073 Å
*a* = 9.5847 (18) Å	Cell parameters from 3529 reflections
*b* = 7.4498 (15) Å	θ = 2.2–27.8°
*c* = 17.599 (4) Å	µ = 0.49 mm^−^^1^
β = 102.997 (4)°	*T* = 100 K
*V* = 1224.5 (4) Å^3^	Plank, colourless
*Z* = 2	0.24 × 0.09 × 0.04 mm

### µ-4,5-Dihydroxybenzene-1,3-disulfonato-bis[aqualithium(I)] hemihydrate (LiTiron) Data collection

**Table:**

Bruker APEXII diffractometer	2851 independent reflections
Radiation source: sealed tube	2313 reflections with *I* > 2σ(*I*)
Graphite monochromator	*R*_int_ = 0.049
Detector resolution: 8.333 pixels mm^-1^	θ_max_ = 27.9°, θ_min_ = 2.2°
ω and φ scans	*h* = −12→12
Absorption correction: multi-scan (SADABS; Bruker, 2014)	*k* = −9→9
*T*_min_ = 0.670, *T*_max_ = 0.746	*l* = −23→22
15864 measured reflections	

### µ-4,5-Dihydroxybenzene-1,3-disulfonato-bis[aqualithium(I)] hemihydrate (LiTiron) Refinement

**Table:**

Refinement on *F*^2^	18 restraints
Least-squares matrix: full	Hydrogen site location: mixed
*R*[*F*^2^ > 2σ(*F*^2^)] = 0.041	H-atom parameters constrained
*wR*(*F*^2^) = 0.100	*w* = 1/[σ^2^(*F*_o_^2^) + (0.0437*P*)^2^ + 1.2637*P*] where *P* = (*F*_o_^2^ + 2*F*_c_^2^)/3
*S* = 1.02	(Δ/σ)_max_ < 0.001
2851 reflections	Δρ_max_ = 0.44 e Å^−^^3^
217 parameters	Δρ_min_ = −0.48 e Å^−^^3^

### µ-4,5-Dihydroxybenzene-1,3-disulfonato-bis[aqualithium(I)] hemihydrate (LiTiron) Special details

**Table:**

Geometry. All esds (except the esd in the dihedral angle between two l.s. planes) are estimated using the full covariance matrix. The cell esds are taken into account individually in the estimation of esds in distances, angles and torsion angles; correlations between esds in cell parameters are only used when they are defined by crystal symmetry. An approximate (isotropic) treatment of cell esds is used for estimating esds involving l.s. planes.

### µ-4,5-Dihydroxybenzene-1,3-disulfonato-bis[aqualithium(I)] hemihydrate (LiTiron) Fractional atomic coordinates and isotropic or equivalent isotropic displacement parameters (Å^2^)

**Table:**

	*x*	*y*	*z*	*U*_iso_*/*U*_eq_	Occ. (<1)
S1	0.32410 (6)	0.37552 (7)	0.67254 (3)	0.01367 (14)	
S2	0.20642 (6)	0.24170 (8)	0.35997 (3)	0.01575 (15)	
O1	0.5203 (2)	0.1795 (3)	0.38983 (11)	0.0257 (5)	0.892 (3)
H1	0.610071	0.172739	0.400448	0.039*	0.892 (3)
O1A	0.6234 (16)	0.305 (2)	0.6540 (9)	0.0257 (5)	0.108 (3)
H1A	0.616503	0.223206	0.685965	0.039*	0.108 (3)
O2	0.71321 (17)	0.2031 (3)	0.52616 (10)	0.0267 (4)	
H2	0.763939	0.215385	0.571438	0.040*	
O3	0.32479 (17)	0.2061 (2)	0.71493 (9)	0.0185 (4)	
O4	0.17924 (16)	0.4482 (2)	0.64770 (9)	0.0173 (3)	
O5	0.42396 (17)	0.5036 (2)	0.71657 (9)	0.0215 (4)	
O6	0.06889 (17)	0.2875 (2)	0.37599 (10)	0.0221 (4)	
O7	0.2162 (2)	0.0587 (3)	0.33493 (11)	0.0390 (5)	
O8	0.24746 (18)	0.3710 (3)	0.30624 (10)	0.0309 (5)	
O9	−0.21479 (18)	0.1015 (2)	0.37158 (10)	0.0233 (4)	
H9A	−0.241410	0.013821	0.342640	0.044*	
H9B	−0.166840	0.058560	0.410700	0.044*	
O11	0.4064 (3)	0.1930 (4)	0.1751 (3)	0.0238 (11)	0.751 (12)
H11A	0.487329	0.164550	0.168051	0.025*	0.751 (12)
H11B	0.366160	0.103101	0.187910	0.025*	0.751 (12)
O11A	0.4377 (10)	0.1619 (13)	0.2239 (10)	0.028 (3)	0.249 (12)
H11C	0.396660	0.068010	0.203321	0.034*	0.249 (12)
H11D	0.483500	0.127750	0.269549	0.034*	0.249 (12)
C1	0.4755 (2)	0.2263 (3)	0.45422 (13)	0.0154 (5)	
H1B	0.506816	0.192678	0.408715	0.018*	0.108 (3)
C2	0.5738 (2)	0.2393 (3)	0.52645 (14)	0.0175 (5)	
C3	0.5268 (2)	0.2844 (3)	0.59252 (14)	0.0169 (5)	
H3	0.592517	0.288774	0.641732	0.020*	0.892 (3)
C4	0.3821 (2)	0.3237 (3)	0.58686 (13)	0.0138 (4)	
C5	0.2839 (2)	0.3139 (3)	0.51595 (13)	0.0134 (4)	
H5	0.185883	0.341503	0.512466	0.016*	
C6	0.3312 (2)	0.2633 (3)	0.45013 (13)	0.0136 (4)	
Li1	−0.1315 (4)	0.3045 (5)	0.3289 (2)	0.0188 (8)	
Li2	0.3913 (4)	0.3966 (6)	0.2447 (2)	0.0224 (9)	
O10	0.011 (6)	−0.038 (9)	0.478 (3)	0.045 (5)	0.20 (5)
H10A	0.057700	−0.123691	0.468720	0.068*	0.20 (5)
H10B	0.066210	0.054340	0.453980	0.068*	0.20 (5)
O10A	0.005 (4)	0.042 (6)	0.507 (3)	0.043 (5)	0.30 (5)
H10C	−0.047541	0.116070	0.529981	0.065*	0.30 (5)
H10D	0.045200	0.118550	0.481750	0.065*	0.30 (5)

### µ-4,5-Dihydroxybenzene-1,3-disulfonato-bis[aqualithium(I)] hemihydrate (LiTiron) Atomic displacement parameters (Å^2^)

**Table:**

	*U*^11^	*U*^22^	*U*^33^	*U*^12^	*U*^13^	*U*^23^
S1	0.0148 (3)	0.0123 (3)	0.0136 (3)	−0.0003 (2)	0.00248 (19)	−0.0012 (2)
S2	0.0166 (3)	0.0155 (3)	0.0143 (3)	0.0031 (2)	0.0016 (2)	−0.0006 (2)
O1	0.0209 (10)	0.0374 (12)	0.0215 (10)	0.0025 (8)	0.0105 (8)	−0.0048 (9)
O1A	0.0209 (10)	0.0374 (12)	0.0215 (10)	0.0025 (8)	0.0105 (8)	−0.0048 (9)
O2	0.0141 (8)	0.0442 (12)	0.0219 (9)	0.0071 (7)	0.0043 (7)	−0.0002 (8)
O3	0.0265 (9)	0.0134 (8)	0.0154 (8)	0.0007 (6)	0.0044 (6)	0.0024 (6)
O4	0.0166 (8)	0.0183 (8)	0.0169 (8)	0.0014 (6)	0.0035 (6)	−0.0019 (7)
O5	0.0208 (9)	0.0214 (9)	0.0218 (9)	−0.0054 (7)	0.0035 (7)	−0.0074 (7)
O6	0.0158 (8)	0.0316 (10)	0.0187 (9)	−0.0009 (7)	0.0034 (6)	−0.0021 (7)
O7	0.0451 (12)	0.0210 (10)	0.0378 (12)	0.0131 (8)	−0.0185 (9)	−0.0163 (9)
O8	0.0236 (9)	0.0469 (12)	0.0239 (10)	0.0088 (8)	0.0085 (7)	0.0187 (9)
O9	0.0293 (9)	0.0184 (9)	0.0220 (9)	−0.0009 (7)	0.0051 (7)	0.0004 (7)
O11	0.0168 (13)	0.0183 (13)	0.037 (3)	−0.0016 (9)	0.0070 (14)	−0.0075 (14)
O11A	0.023 (4)	0.025 (4)	0.036 (8)	−0.004 (3)	0.007 (4)	−0.011 (4)
C1	0.0183 (11)	0.0116 (11)	0.0182 (12)	0.0011 (8)	0.0082 (9)	0.0009 (9)
C2	0.0121 (10)	0.0165 (11)	0.0245 (13)	0.0023 (8)	0.0052 (9)	0.0012 (10)
C3	0.0157 (11)	0.0151 (12)	0.0188 (12)	−0.0001 (8)	0.0014 (9)	0.0005 (9)
C4	0.0172 (11)	0.0092 (10)	0.0154 (11)	−0.0010 (8)	0.0046 (8)	0.0009 (8)
C5	0.0130 (10)	0.0107 (10)	0.0168 (11)	0.0003 (8)	0.0042 (8)	−0.0009 (9)
C6	0.0160 (11)	0.0110 (10)	0.0134 (11)	−0.0017 (8)	0.0023 (8)	0.0009 (8)
Li1	0.021 (2)	0.0144 (19)	0.019 (2)	−0.0001 (15)	0.0005 (15)	0.0006 (16)
Li2	0.020 (2)	0.022 (2)	0.025 (2)	−0.0026 (16)	0.0045 (16)	0.0007 (17)
O10	0.042 (8)	0.050 (6)	0.049 (13)	0.010 (7)	0.023 (8)	0.020 (10)
O10A	0.040 (7)	0.050 (6)	0.047 (12)	0.008 (6)	0.024 (7)	0.020 (9)

### µ-4,5-Dihydroxybenzene-1,3-disulfonato-bis[aqualithium(I)] hemihydrate (LiTiron) Geometric parameters (Å, º)

**Table:**

S1—O3	1.4655 (17)	O9—H9B	0.8033 (17)
S1—O4	1.4626 (16)	O9—Li1	1.939 (4)
S1—O5	1.4466 (16)	O11—H11A	0.840 (2)
S1—C4	1.764 (2)	O11—H11B	0.829 (3)
S1—Li1^i^	3.012 (4)	O11—Li2	1.975 (5)
S1—Li1^ii^	3.005 (4)	O11A—H11C	0.843 (8)
S2—O6	1.4493 (17)	O11A—H11D	0.862 (18)
S2—O7	1.4422 (19)	O11A—Li2	1.861 (9)
S2—O8	1.4642 (19)	C1—H1B	0.9500
S2—C6	1.766 (2)	C1—C2	1.405 (3)
O1—H1	0.8400	C1—C6	1.396 (3)
O1—C1	1.345 (3)	C2—C3	1.380 (3)
O1A—H1A	0.8400	C3—H3	0.9500
O1A—C3	1.265 (16)	C3—C4	1.398 (3)
O2—H2	0.8400	C4—C5	1.386 (3)
O2—C2	1.364 (3)	C5—H5	0.9500
O3—Li1^ii^	1.957 (4)	C5—C6	1.388 (3)
O4—Li1^i^	1.964 (4)	Li2—H11D	2.193 (4)
O5—Li2^iii^	1.900 (4)	O10—H10A	0.81 (6)
O6—Li1	1.917 (4)	O10—H10B	1.02 (6)
O7—Li2^iv^	1.958 (5)	O10A—H10C	0.90 (5)
O8—Li2	1.945 (5)	O10A—H10D	0.86 (4)
O9—H9A	0.8323 (17)		
			
O3—S1—C4	106.28 (10)	O2—C2—C3	123.8 (2)
O3—S1—Li1^i^	128.22 (11)	C3—C2—C1	120.0 (2)
O4—S1—O3	111.49 (10)	O1A—C3—C2	115.8 (7)
O4—S1—C4	106.64 (10)	O1A—C3—C4	124.0 (7)
O4—S1—Li1^ii^	111.62 (10)	C2—C3—H3	120.1
O5—S1—O3	111.63 (10)	C2—C3—C4	119.8 (2)
O5—S1—O4	112.66 (10)	C4—C3—H3	120.1
O5—S1—C4	107.73 (10)	C3—C4—S1	118.89 (17)
C4—S1—Li1^i^	118.47 (11)	C5—C4—S1	120.01 (16)
C4—S1—Li1^ii^	132.73 (11)	C5—C4—C3	121.0 (2)
Li1^ii^—S1—Li1^i^	108.75 (7)	C4—C5—H5	120.6
O6—S2—O8	111.04 (10)	C4—C5—C6	118.8 (2)
O6—S2—C6	105.40 (10)	C6—C5—H5	120.6
O7—S2—O6	113.98 (12)	C1—C6—S2	119.42 (17)
O7—S2—O8	112.28 (13)	C5—C6—S2	119.48 (17)
O7—S2—C6	106.44 (10)	C5—C6—C1	121.1 (2)
O8—S2—C6	107.11 (11)	S1^v^—Li1—S1^i^	112.65 (12)
C1—O1—H1	109.5	O3^v^—Li1—S1^i^	91.92 (14)
C3—O1A—H1A	109.5	O3^v^—Li1—O4^i^	104.25 (19)
C2—O2—H2	109.5	O4^i^—Li1—S1^v^	128.23 (18)
S1—O3—Li1^ii^	122.14 (15)	O6—Li1—S1^i^	127.50 (18)
S1—O4—Li1^i^	122.34 (15)	O6—Li1—S1^v^	106.74 (17)
S1—O5—Li2^iii^	154.32 (17)	O6—Li1—O3^v^	113.8 (2)
S2—O6—Li1	142.95 (17)	O6—Li1—O4^i^	103.28 (19)
S2—O7—Li2^iv^	137.88 (16)	O6—Li1—O9	103.95 (19)
S2—O8—Li2	138.70 (17)	O9—Li1—S1^i^	108.40 (17)
H9A—O9—H9B	104.44 (18)	O9—Li1—S1^v^	91.18 (15)
Li1—O9—H9A	118.14 (19)	O9—Li1—O3^v^	110.7 (2)
Li1—O9—H9B	115.87 (18)	O9—Li1—O4^i^	121.0 (2)
H11A—O11—H11B	109.7 (3)	O5^iii^—Li2—O7^vi^	108.3 (2)
Li2—O11—H11A	119.0 (3)	O5^iii^—Li2—O8	124.0 (2)
Li2—O11—H11B	110.4 (4)	O5^iii^—Li2—O11	109.3 (2)
H11C—O11A—H11D	104.3 (14)	O7^vi^—Li2—O11	97.5 (2)
Li2—O11A—H11C	139.0 (10)	O8—Li2—O7^vi^	97.7 (2)
Li2—O11A—H11D	100.9 (8)	O8—Li2—O11	115.5 (2)
O1—C1—C2	120.3 (2)	O11A—Li2—O5^iii^	101.2 (3)
O1—C1—C6	120.5 (2)	O11A—Li2—O7^vi^	123.3 (6)
C2—C1—H1B	120.4	O11A—Li2—O8	104.4 (4)
C6—C1—H1B	120.4	H10A—O10—H10B	95 (5)
C6—C1—C2	119.2 (2)	H10C—O10A—H10D	101 (4)
O2—C2—C1	116.2 (2)		
			
S1—C4—C5—C6	−176.51 (16)	O8—S2—O6—Li1	66.9 (3)
O1—C1—C2—O2	0.3 (3)	O8—S2—O7—Li2^iv^	−74.8 (3)
O1—C1—C2—C3	−178.9 (2)	O8—S2—C6—C1	−62.2 (2)
O1—C1—C6—S2	1.9 (3)	O8—S2—C6—C5	118.80 (18)
O1—C1—C6—C5	−179.2 (2)	C1—C2—C3—O1A	−175.5 (9)
O1A—C3—C4—S1	−9.0 (10)	C1—C2—C3—C4	−2.4 (3)
O1A—C3—C4—C5	174.0 (10)	C2—C1—C6—S2	−178.43 (17)
O2—C2—C3—O1A	5.3 (10)	C2—C1—C6—C5	0.5 (3)
O2—C2—C3—C4	178.4 (2)	C2—C3—C4—S1	178.46 (17)
O3—S1—O4—Li1^i^	127.63 (18)	C2—C3—C4—C5	1.5 (3)
O3—S1—O5—Li2^iii^	79.3 (4)	C3—C4—C5—C6	0.4 (3)
O3—S1—C4—C3	−73.21 (19)	C4—S1—O3—Li1^ii^	147.14 (17)
O3—S1—C4—C5	103.75 (19)	C4—S1—O4—Li1^i^	−116.77 (18)
O4—S1—O3—Li1^ii^	−97.05 (18)	C4—S1—O5—Li2^iii^	−37.0 (4)
O4—S1—O5—Li2^iii^	−154.4 (4)	C4—C5—C6—S2	177.56 (17)
O4—S1—C4—C3	167.74 (17)	C4—C5—C6—C1	−1.4 (3)
O4—S1—C4—C5	−15.3 (2)	C6—S2—O6—Li1	−177.4 (3)
O5—S1—O3—Li1^ii^	29.9 (2)	C6—S2—O7—Li2^iv^	168.3 (3)
O5—S1—O4—Li1^i^	1.2 (2)	C6—S2—O8—Li2	76.1 (3)
O5—S1—C4—C3	46.6 (2)	C6—C1—C2—O2	−179.4 (2)
O5—S1—C4—C5	−136.48 (18)	C6—C1—C2—C3	1.4 (3)
O6—S2—O7—Li2^iv^	52.6 (3)	Li1^i^—S1—O3—Li1^ii^	−63.3 (2)
O6—S2—O8—Li2	−169.3 (2)	Li1^ii^—S1—O4—Li1^i^	91.57 (15)
O6—S2—C6—C1	179.47 (17)	Li1^ii^—S1—O5—Li2^iii^	95.4 (4)
O6—S2—C6—C5	0.5 (2)	Li1^i^—S1—O5—Li2^iii^	−153.7 (4)
O7—S2—O6—Li1	−61.1 (3)	Li1^i^—S1—C4—C3	133.71 (18)
O7—S2—O8—Li2	−40.4 (3)	Li1^ii^—S1—C4—C3	−49.2 (2)
O7—S2—C6—C1	58.1 (2)	Li1^i^—S1—C4—C5	−49.3 (2)
O7—S2—C6—C5	−120.90 (19)	Li1^ii^—S1—C4—C5	127.79 (19)

Symmetry codes: (i) −*x*, −*y*+1, −*z*+1; (ii) *x*+1/2, −*y*+1/2, *z*+1/2; (iii) −*x*+1, −*y*+1, −*z*+1; (iv) −*x*+1/2, *y*−1/2, −*z*+1/2; (v) *x*−1/2, −*y*+1/2, *z*−1/2; (vi) −*x*+1/2, *y*+1/2, −*z*+1/2.

### µ-4,5-Dihydroxybenzene-1,3-disulfonato-bis[aqualithium(I)] hemihydrate (LiTiron) Hydrogen-bond geometry (Å, º)

**Table:**

*D*—H···*A*	*D*—H	H···*A*	*D*···*A*	*D*—H···*A*
O1—H1···O2	0.84	2.22	2.685 (3)	115
O1—H1···O9^vii^	0.84	1.93	2.693 (3)	150
O1*A*—H1*A*···O8^ii^	0.84	2.31	2.978 (17)	136
O2—H2···O1*A*	0.84	2.29	2.693 (16)	110
O2—H2···O7^viii^	0.84	2.60	3.082 (3)	117
O2—H2···O11^ii^	0.84	2.13	2.952 (6)	166
O9—H9*A*···O3^ix^	0.8323 (17)	1.9961 (16)	2.821 (2)	170.86 (13)
O9—H9*B*···O10^ix^	0.8033 (17)	2.19 (6)	2.96 (6)	160.0 (18)
O9—H9*B*···O10	0.8033 (17)	1.98 (6)	2.73 (6)	155.0 (16)
O9—H9*B*···O10*A*^ix^	0.8033 (17)	2.02 (4)	2.80 (4)	164.8 (14)
O9—H9*B*···O10*A*	0.8033 (17)	2.08 (4)	2.83 (4)	156.2 (12)
O11—H11*A*···O4^x^	0.840 (2)	2.1244 (16)	2.957 (3)	171.19 (17)
O11—H11*B*···O8^iv^	0.829 (3)	2.0583 (19)	2.873 (3)	167.4 (4)
O11*A*—H11*C*···O6^iv^	0.843 (8)	2.5753 (18)	3.290 (8)	143.3 (5)
O11*A*—H11*C*···O8^iv^	0.843 (8)	1.9964 (18)	2.776 (8)	153.2 (8)
O11*A*—H11*D*···O1	0.862 (18)	2.1019 (19)	2.851 (17)	145.0 (5)
O10—H10*A*···O2^viii^	0.81 (6)	2.2562 (16)	2.93 (6)	141 (4)
O10—H10*B*···O6	1.02 (6)	2.2175 (18)	3.14 (6)	150 (3)
O10*A*—H10*C*···O2^xi^	0.90 (5)	2.3694 (16)	3.13 (5)	142 (3)
O10*A*—H10*D*···O6	0.86 (4)	2.3000 (17)	3.10 (5)	155 (3)

Symmetry codes: (ii) *x*+1/2, −*y*+1/2, *z*+1/2; (iv) −*x*+1/2, *y*−1/2, −*z*+1/2; (vii) *x*+1, *y*, *z*; (viii) −*x*+1, −*y*, −*z*+1; (ix) −*x*, −*y*, −*z*+1; (x) *x*+1/2, −*y*+1/2, *z*−1/2; (xi) *x*−1, *y*, *z*.

## References

[bb1] Boukhalfa, H. & Crumbliss, A. L. (2002). *Biometals*, **15**, 325–339. 10.1023/a:1020218608266 12405526

[bb2] Bruker (2014). *APEX2*, *SAINT* and *SADABS.* Bruker AXS Inc, Madison, Wisconsin, USA.

[bb3] Bruker (2016). *SAINT*. Bruker AXS Inc, Madison, Wisconsin, USA.

[bb4] Côté, A. P. & Shimizu, G. K. H. (2001). *Chem. Commun.* pp. 251–252.

[bb5] Côté, A. P. & Shimizu, G. K. H. (2003). *Chem. Eur. J.* **9**, 5361–5370. 10.1002/chem.200305102 14613146

[bb6] Dolomanov, O. V., Bourhis, L. J., Gildea, R. J., Howard, J. A. K. & Puschmann, H. (2009). *J. Appl. Cryst.* **42**, 339–341.

[bb7] Groom, C. R., Bruno, I. J., Lightfoot, M. P. & Ward, S. C. (2016). *Acta Cryst.* B**72**, 171–179. 10.1107/S2052520616003954 PMC4822653 27048719

[bb8] Guan, L. (2016). *Synth. React. Inorg. Met.-Org. Nano-Met. Chem.* **46**, 1–5.

[bb9] Guan, L. & Wang, Y. (2016). *J. Coord. Chem.* **69**, 3107–3114.

[bb10] Guan, L. & Wang, Y. (2017). *J. Coord. Chem.* **70**, 2520–2529.

[bb11] Lee, B. P., Messersmith, P. B., Israelachvili, J. N. & Waite, J. H. (2011). *Annual Review of Materials Research*, Vol 41, edited by D. R. Clarke & P. Fratzl, pp. 99–132. Palo Alto: Annual Reviews. 10.1146/annurev-matsci-062910-100429 PMC3207216 22058660

[bb12] Loomis, L. D. & Raymond, K. N. (1991). *Inorg. Chem.* **30**, 906–911.

[bb13] Lu, L., Jun, W., Wei-Ping, W., Xiu-Lan, Z. & Bin, X. (2014). *Synth. React. Inorg. Met.-Org. Nano-Met. Chem.* **44**, 393–396.

[bb14] Pierpont, C. G. & Lange, C. W. (1994). *Progress in Inorganic Chemistry*, Vol 41, edited by K. D. Karlin, pp. 331–442. New York: John Wiley & Sons Inc.

[bb15] Rapp, M. V., Maier, G. P., Dobbs, H. A., Higdon, N. J., Waite, J. H., Butler, A. & Israelachvili, J. N. (2016). *J. Am. Chem. Soc.* **138**, 9013–9016. 10.1021/jacs.6b03453 27415839

[bb16] Raymond, K. N., Allred, B. E. & Sia, A. K. (2015). *Acc. Chem. Res.* **48**, 2496–2505. 10.1021/acs.accounts.5b00301 PMC4576731 26332443

[bb17] Riley, P. E., Haddad, S. F. & Raymond, K. N. (1983). *Inorg. Chem.* **22**, 3090–3096.

[bb18] Sever, M. J. & Wilker, J. J. (2004). *Dalton Trans.* pp. 1061–1072. 10.1039/b315811j 15252685

[bb19] Sheldrick, G. M. (2008). *Acta Cryst.* A**64**, 112–122. 10.1107/S0108767307043930 18156677

[bb20] Sheldrick, G. M. (2015). *Acta Cryst.* C**71**, 3–8.

[bb21] Sheriff, T. S., Carr, P. & Piggott, B. (2003). *Inorg. Chim. Acta*, **348**, 115–122.

[bb22] Sommer, L. (1963*a*). *Collect. Czech. Chem. Commun.* **28**, 2102–2130.

[bb23] Sommer, L. (1963*b*). *Z. Anorg. Allg. Chem.* **321**, 191–197.

[bb24] Springer, S. D. & Butler, A. (2016). *Coord. Chem. Rev.* **306**, 628–635.

[bb25] Sugumaran, M. & Robinson, W. E. (2012). *Comp. Biochem. Physiol. Part B: Biochem. Mol. Biol.* **163**, 1–25. 10.1016/j.cbpb.2012.05.005 22580032

[bb26] Wang, W. G., Zhang, J., Ju, Z. F. & Song, L. J. (2005). *Appl. Organomet. Chem.* **19**, 191–192.

[bb27] Yang, B., Hoober-Burkhardt, L., Wang, F., Surya Prakash, G. K. & Narayanan, S. R. (2014). *J. Electrochem. Soc.* **161**, A1371–A1380.

[bb28] Yoe, J. H. & Armstrong, A. R. (1945). *Science*, **102**, 207–207. 10.1126/science.102.2643.207 17787144

[bb29] Yoe, J. H. & Armstrong, A. R. (1947). *Anal. Chem.* **19**, 100–102.

[bb30] Zhang, X., Ge, C., Guan, L. & Sun, Z. (2008). *Acta Cryst.* E**64**, m396–m397. 10.1107/S1600536807067918 PMC2960182 21201347

